# Choking under pressure: Does it get easier with age? How loneliness affects social monitoring across the life span

**DOI:** 10.1177/0165025420979369

**Published:** 2020-12-20

**Authors:** Ellie Pearce, Manuela Barreto, Christina Victor, Claudia Hammond, Alice M. Eccles, Matthew T. Richins, Alisha O’Neil, Megan L. Knowles, Pamela Qualter

**Affiliations:** 1 University College London, UK; 2 University of Exeter, UK; 3 Brunel University, London, UK; 4 BBC Radio 4, UK; 5 University of Manchester, UK; 6 Franklin and Marshall College, USA

**Keywords:** Cognitive bias, emotion recognition, loneliness, social cognitions, social skills

## Abstract

Previous experimental work showed that young adults reporting loneliness performed less well on emotion recognition tasks (Diagnostic Analysis of Nonverbal Accuracy [DANVA-2]) if they were framed as indicators of social aptitude, but not when the same tasks were framed as indexing academic aptitude. Such findings suggested that undergraduates reporting loneliness possessed the social monitoring skills necessary to read the emotions underlying others’ facial expressions, but that they choked under social pressure. It has also been found that undergraduates reporting loneliness have better recall for both positive and negative social information than their non-lonely counterparts. Whether those effects are evident across different age groups has not been examined. Using data from the British Broadcasting Corporation (BBC) Loneliness Experiment that included participants aged 16–99 years (*N* = 54,060), we (i) test for replication in a larger worldwide sample and (ii) extend those linear model analyses to other age groups. We found only effects for participants aged 25–34 years: In this age group, loneliness was associated with increased recall of negative individual information, and with choking under social pressure during the emotion recognition task; those effects were small. We did not find any such effects among participants in other age groups. Our findings suggest that different cognitive processes may be associated with loneliness in different age groups, highlighting the importance of life-course approaches in this area.

Loneliness is associated with heightened social monitoring (Spithoven et al., 2017). For instance, children and adolescents who report loneliness are more attentive to potential social threat during eye-tracking tasks than their non-lonely peers (Bangee et al., 2014; Qualter et al., 2013), and lonely adolescents are more sensitive in their detection of facial expressions of sadness and fear compared to non-lonely controls (Vanhalst et al., 2017). Moreover, lonely young adults have (i) better recall of social information compared to their non-lonely peers (Gardner et al., 2005) and (ii) their brains respond faster to signs of potential rejection in the social environment (Cacioppo et al., 2016). However, the majority of previous studies in this area have used relatively small samples drawn predominantly from undergraduate student populations. Consequently, there is a need for this work to be developed using larger samples representing other demographic groups, to explore whether there is consistency in associations between social monitoring and loneliness across development (Qualter, Vanhalst, et al., 2015). The British Broadcasting Corporation (BBC) Loneliness Experiment data provides such an opportunity: It comprises a worldwide sample several orders of magnitude larger than those previously used, which is drawn from the general population and has an age range of 16–99 years. Using that data set, we attempt to replicate and extend previous results pertaining to social monitoring and loneliness, exploring whether (i) loneliness is associated with enhanced social memory and (ii) there is a social framing effect on emotion recognition (ER) in facial expressions among those who report loneliness.

## Loneliness and Social Monitoring: The Theory

Humans have a strong desire for social connectedness (Baumeister & Leary, 1995), which is fulfilled by affiliation with and acceptance from others. Humans devote considerable effort to understand and negotiate social interactions and relationships; to avoid exclusion, which ultimately leads to fewer resources, individuals are motivated to monitor and regulate their levels of social inclusion (Gardner et al., 2005). Gardner et al. argue that when individuals do not feel connected—when they experience loneliness—the social monitoring system is engaged, providing social information that promotes inclusion or enables avoidance of potentially rejecting social situations. Loneliness is theorized to be part of an innate motivational drive that maintains social contact and prevents the aversive consequences of isolation: The negative emotions that accompany loneliness activate an innate social monitoring system that increases attentiveness to social information, prioritizing it over nonsocial information, with the aim of improving social connection (Qualter, Vanhalst et al., 2015).

In their evolutionary theory of the origins of loneliness, Cacioppo and Hawkley (2009) also argued that lonely individuals attend to social information. They argued that, while motivation to re-affiliate is high when people report loneliness, we should expect more attention to be given to negative social information than positive social information because individuals will want to avoid social exclusion, which puts them on the periphery of the social group and, in evolutionary terms, makes them vulnerable to predator threats. The thesis is that loneliness increases social vigilance, specifically to negative social stimuli through its activation of an innate self-preservation mechanism (Matthews & Tye, 2019).

The argument being put forward by Gardner et al. (2005) and Cacioppo and Hawkley (2009) is that when social monitoring is activated during loneliness, the salience of interpersonal information in facial expressions or in tone of voice is heightened. This, in turn, is expected to result in more accurate encoding and processing of social information, which should lead to social reconnection. Gardner argued that lonelier individuals have heightened interpersonal sensitivity to positive and negative social cues and can more accurately decode verbal and nonverbal social cues; Cacioppo and Hawkley argued that attention would be more focused on negative social cues, reflective of potential threat.

## Is Loneliness associated with Social Monitoring?

So, is there empirical evidence that loneliness is associated with enhanced social monitoring? Evidence shows that attention to social information (both positive and negative) increases with loneliness (Gardner et al., 2005; Knowles et al., 2015). Lonely people also detect and respond to social information quicker than their non-lonely peers (Cacioppo et al., 2015; Cacioppo et al., 2016), but the social monitoring of individuals feeling lonely focused more on potential threats than on affiliative cues. And, while the time-course of attention to social stimuli among individuals reporting loneliness varies by developmental stage (Qualter, Vanhalst, et al., 2015), loneliness appears to be associated with a preference for social versus nonsocial information and heightened attention to social threats among the young adults and adolescents taking part in those studies (Spithoven et al., 2017).

Processing social information for affiliative or threat potential requires individuals to also perceive accurately the emotions in faces and voices of others in the social environment, as well as to anticipate how those are linked to subsequent behavior. Researchers exploring accuracy of ER and loneliness have produced mixed findings, with loneliness associated with increased facial ER accuracy (Gardner et al., 2005), decreased facial ER accuracy (Zysberg, 2012), or not associated at all (Kanai et al., 2012; Lodder et al., 2016). Other work found that ER ability predicted reductions in loneliness over time among adolescents (Wols et al., 2015). In those studies, the recognition of positive and negative emotions were not examined separately. Where emotion-specific effects have been explored, findings, again, are inconsistent, with loneliness shown to be associated with increased recognition of angry faces, but not fearful, sad, or happy faces (Lodder et al., 2016), or of sadness and fear, but not anger (Vanhalst et al., 2017); findings also show increased attention to angry faces in a crowd (Bangee & Qualter, 2018). To our knowledge, only two studies have examined the association between loneliness and vocal ER (Knowles et al., 2015; Morningstar et al., 2019). Only Morningstar et al. explored emotion specificity, finding that adolescents reporting loneliness had better recognition of friendliness compared to their non-lonely peers, suggesting that people reporting loneliness may be attuned to affiliative cues in affective prosody.

## Choking under Pressure

If lonely individuals show enhanced social monitoring, which should increase successful social reconnection, one might wonder why, then, some people reporting loneliness do not escape from their unpleasant state of disconnection. How can the extant findings be reconciled with this painful reality? One possibility is that individuals who perceive they have a deficit in their social relations are highly motivated to seek (re)connection, creating (self-generated) pressure for them to perform well in social situations. That supposition led to the ‘choking under pressure’ hypothesis of loneliness (Knowles et al., 2015). The hypothesis proposes that people reporting higher loneliness are attuned to social information (greater social monitoring), but choke under the pressure of their strong desire for connection: They underperform on social tasks due to distraction and overmonitoring of the situation and their performance. That might also involve a feedback-loop in which those individuals reporting loneliness expect to be poor at social tasks as an explanation for feeling a lack of connection and, thus, misinterpret cues (emotions in faces and voices) that would aid them in interpersonal reconnection. The idea that loneliness can lead to choking under pressure is also evident in Cacioppo and Hawkley’s (2009) evolutionary model of loneliness. They propose that prolonged use of hypervigilance toward social threat can negatively impact performance because the person is too self-focused, usually on their own anxiety, that they fail to regain connection (Qualter, Vanhalst, et al., 2015).

As an initial test of their hypothesis, Knowles et al. (2015) framed various tasks relevant to social monitoring as either pertaining to social skills or general intelligence and tested whether undergraduates reporting loneliness differed from their peers in how well they performed those tasks depending on whether they perceived they were being judged on their social skills or not. In support of the choking hypothesis, Knowles et al. found significant interactions between degree of loneliness and framing condition on the performance of tasks involving identification of emotions from images of faces using the Diagnostic Analysis of Nonverbal Accuracy, DANVA-2, measure (Study 1) or vocal tones (Study 2). When social monitoring tasks were framed as socially diagnostic, undergraduates reporting loneliness performed significantly worse than their peers; when the task was apparently diagnostic of nonsocial abilities, there was a trend toward better performance among individuals reporting loneliness, indicating that the necessary ER skills were intact.

Evidence supports the notion that loneliness increases social monitoring, particularly of social cues signaling potential exclusion/threat. Knowles et al (2015) provided evidence for the choking under pressure phenomenon in relation to loneliness, such that, despite both ability and motivation, performance on a social task is poor. However, the idea that loneliness is associated with social monitoring and that those reporting loneliness might choke under pressure has yet to be replicated with larger samples. The primary aim of the current study was to test whether those findings hold in a larger and more diverse sample.

Given that most previous studies on social monitoring and loneliness were conducted with undergraduate students ranging from 18 years to 22 years of age, it is as yet unclear how generalizable the findings are to other demographic groups. Indeed, there is some evidence that social monitoring processes in relation to loneliness may differ between age groups (Bangee et al., 2014; Morningstar et al., 2020; Qualter et al., 2013), but there is comparatively little work examining social monitoring among middle-age and older-age adults (Qualter, Vanhalst, et al., 2015; Spithoven et al., 2017). Researchers have urged the research community to replicate prior work in samples that represent the entire life span (Böger & Huxhold, 2018; Luhmann & Hawkley, 2016). In fact, Knowles et al. (2015) suggested that older individuals may be less susceptible to the choking effect than the undergraduate population they recruited from: They suggested that older individuals might be more likely to attribute their loneliness to age-related or life factors (such as bereavement, moving jobs, parenthood, retirement) rather than their social skills or performance. Older individuals are also more used to the experience of loneliness and know how best to cope with it. Moreover, younger individuals may be more prone to choke as they might be more likely to interact with new, unfamiliar others, particularly in the university context. In addition, both social and personal identities are likely to be more established among older people, which means that feeling part of a peer group is less important than it is for younger cohorts (Qualter, Vanhalst, et al., 2015). That means older people, consequently, might feel less pressure to perform well in social situations and to curate a positive social reputation.

In the current article, using data from the BBC Loneliness Experiment, we build on previous work to examine social monitoring among people reporting loneliness and their non-lonely peers across the life span. We first tested for a replication of the findings on social monitoring reported by Gardner et al. (2005; Study 1), where undergraduates who reported higher loneliness were found to have better recall of social information provided in diary excerpts compared to their peers. In that study, loneliness was related to greater recall for both positive and negative social (collective and interpersonal) events. Following Gardner, we expected to find that reporting loneliness was associated with greater incidental recall of social events. Thus, we hypothesized that loneliness would be positively associated with performance on the social memory tasks: Lonelier individuals would recall a greater number of events from the diary entries when they relate to social (interpersonal and collective) events than individual events. We extended the earlier work to examine whether the positive relationship between loneliness and recall of social information holds across ages in a substantially larger sample recruited from the general population.

We also explored the choking under pressure hypothesis using the same DANVA-2 measure and social versus nonsocial framing stimuli (varied experimentally), both for the full set of DANVA-2 faces and for the low intensity (harder to detect) set, following Knowles et al (2015). Based on findings of Knowles and colleagues, we expected to find that individuals reporting loneliness choked under pressure when they were under the impression they were completing a social skills task. Specifically, we hypothesized that loneliness would be negatively associated with performance on ER tasks (lonelier individuals have lower DANVA scores) only when the task is framed as relevant to social skills. We also expected age to moderate that association, such that the choking effect would be evident for young adults but may not be evident for older age-groups where social relationships have become established.

## Method

### Participants

Participants took part in an online survey advertised through the BBC, which, through its world service and website, has a global audience. Accompanying programs were broadcast on Radio 4 and the World Service and the survey link was promoted on the BBC website. People took part from all over the world but were predominantly from Western countries (Online Supplemental Table S1). Participants were asked to provide informed consent before starting the survey. Ethics approval was granted by The University of Manchester UREC Committee (2017-2710-4594).

Due to time limitations, we selectively coded data for 2,771 of the 9,273 participants (30%) who took part in the social memory task, leaving 2,632 participants (female = 1,342 [51%], age *M* = 47.93 years, *SD* = 18.95 years, range = 16–99 years) once those with missing data were excluded (see Online Supplemental Table S2 for details of missingness). For the second study, which explored ER and the choking under pressure phenomenon, the sample comprised 22,054 participants (female = 14,895 [68%], age *M* = 49.57, *SD* = 15.38 years, range = 16–99 years) (see Online Supplemental Table S2 for details of missing data).

#### Procedure

Participants in the BBC Loneliness Experiment completed the UCLA 4-item measure of loneliness (Russell et al., 1980), which asked participants to rate how often they felt (i) a lack of companionship, (ii) left out, (iii) isolated from others, and (iv) in tune with others (reverse scored) on a 5-point scale anchored with 1 meaning “never” and 5 meaning “very often.” Higher scores represent higher loneliness (Cronbach’s α = .845).

Participants in the BBC Loneliness Experiment were presented randomly with one of four experimental tasks, which appeared after they provided basic demographic information at the start of the survey. One of the tasks presented to participants was the *social-memory task* (Gardner et al., 2005). Participants were asked to read four short extracts from a diary for an individual of the same sex as them (as in Gardner et al., 2005). Following a set of other questions in the survey, participants were then asked to list as many events as they could remember from the diary they had read. Those lists were rated by two coders who were blind to the participants’ loneliness scores as (i) individual events that were positive (e.g., I played a chess match and won), (ii) individual events that were negative (e.g., when cycling to the office, I fell off my bike and hurt my head), (iii) interpersonal events that were positive (e.g., I received a parcel from my cousin (who I am very close to) and it was full of hilarious pictures from our last holiday together), (iv) interpersonal events that were negative (e.g., my best friend let me down: we had made plans to do something at the weekend, but I guess it didn’t matter), (v) collective events that were positive (e.g., my office has got cinema tickets for our good productivity results), and (vi) collective events that were negative (e.g., my group entered a poster competition and we just found out we didn’t win a prize). There were four instances of each kind of event, so participants could score a maximum of four correct responses for each (Gardner et al., 2005). Scores for the number of correctly remembered events were summed for each of the six categories. Descriptive statistics for Recall for different event types/valences for this sample can be found in Table 1 and split by Age Group in Online Supplemental Table S3.

**Table 1.** Descriptive Statistics for Male and Female Participants for Memory Task Sample.

		*N*	Minimum	Maximum	Mean	*SD*
		*Males*				
Age		1,291	16.00	99.00	47.18	18.65
Loneliness^a^		1,291	4.00	20.00	11.13	4.61
Recall scores for different types of events	Individual positive	1,291	0.00	3.00	0.35	0.53
	Individual negative	1,291	0.00	3.00	0.31	0.58
	Interpersonal positive	1,291	0.00	3.00	0.19	0.47
	Interpersonal negative	1,291	0.00	4.00	0.33	0.66
	Collective positive	1,291	0.00	3.00	0.15	0.43
	Collective negative	1,291	0.00	4.00	0.21	0.53
		Females				
Age		1,341	16.00	99.00	48.65	19.23
Loneliness (raw scores)		1,341	4.00	20.00	10.43	4.57
Recall scores for different types of events	Individual positive	1,341	0.00	3.00	0.34	0.51
	Individual negative	1,341	0.00	4.00	0.33	0.61
	Interpersonal positive	1,341	0.00	3.00	0.23	0.51
	Interpersonal negative	1,341	0.00	4.00	0.33	0.63
	Collective positive	1,341	0.00	3.00	0.14	0.41
	Collective negative	1,341	0.00	4.00	0.25	0.56

*Note*. *N* = sample size; *SD* = standard deviation.

^a^ Total raw scores on the 4-item UCLA scales, not *z*-scores: possible total scores range from 1–20; participants are asked to rate how often they felt (i) a lack of companionship, (ii) left out, (iii) isolated from others, and (iv) in tune with others (reverse scored) on a 5-point scale anchored from “never” (1) and to “very often” (5). Higher scores on the 4-item UCLA represented higher loneliness (α = .845). In the social memory task, participants read four short extracts from a diary for an individual of the same sex; after reading the diary, they listed as many events as they could remember from the diary. Two coders, blind to the participants’ loneliness scores, categorized responses as (i) individual events that were positive (e.g., I played a chess match and won), (ii) individual events that were negative (e.g., when cycling to the office, I fell off my bike and hurt my head), (iii) interpersonal events that were positive (e.g., I received a parcel from my cousin [who I am very close to] and it was full of hilarious pictures from our last holiday together), (iv) interpersonal events that were negative (e.g., my best friend let me down: we had made plans to do something at the weekend, but I guess it didn’t matter), (v) collective events that were positive (e.g., my office has got cinema tickets for our good productivity results), and (vi) collective events that were negative (e.g., my group entered a poster competition and we just found out we didn’t win a prize). In the diary, there had been four instances of each kind of event, so there was a maximum of four correct responses for each event. Scores for the number of correctly remembered events were summed for each of the six categories.

Different participants were presented with the *ER task* (DANVA-2; Nowicki & Duke, 1994), which was used in both the studies we seek to replicate (Gardner et al., 2005; Knowles et al., 2015). Participants were randomly assigned to either a nonsocial framing condition (*N* = 11,089) or a social framing condition (*N* = 10,965) as used in Knowles et al. (2015); we also used the same phrasing for the social and nonsocial framing conditions as was used in the Knowles et al study as follows.

You should know that people who do well on this task tend to perform well in problem-solving situations every day, and tend to excel in school and attain good jobs after graduation. (nonsocial framing)

You should know that people who do well on this task tend to perform well in social situations every day, and tend to form strong, long-lasting relationships with other people throughout life. (social framing)

The DANVA-2 includes photos of 24 male and female young adult faces, presented individually, for 2 s. The participant is asked to choose which emotion best characterizes the expression of the depicted face, from four options: happy, fearful, angry, or sad. There are six faces for each emotion, and three of those are “low intensity,” where the emotion is subtler and, therefore, harder to detect, than in the three “high-intensity” pictures. Of the 13 female faces, eight show low-intensity emotions (two each for all four emotions), whereas only four of the 11 male faces show low-intensity emotion (one each for all four emotions). For the high-intensity emotions, there are two male faces and one female face for each of the emotions except anger, which is the opposite.

In Knowles et al. (2015), they used both the total DANVA-2 score summed across all 24 faces and the low-intensity only score. To attempt a replication, we also used those scores. We also developed the analyses from the previous studies. Given experimental findings that loneliness makes people more sensitive to threatening/negative emotions than to positive social stimuli, in line with Cacioppo and Hawkley’s evolutionary theory of loneliness, we examined the low-intensity stimuli for the four emotions separately. We expected there might be a framing effect for threatening/negative emotional expressions (fear, anger, sadness), but not positive ones (happiness).

### Analysis Plan

We used RStudio Version 1.1.432 to conduct the analyses and SPSS version 26 to create the tables of descriptive statistics. To make the analyses more manageable and reduce the number of tests being run, we collapsed the seven decade-based age categories from the original data set into four age groups based on life transitions: 16–24 years, 25–34 years, 35–64 years, and 65 years and over. Given multiple testing, we reduced our α cutoff to *p* < .001. We acknowledge that adjustment is conservative, but we wanted to control for Type 1 errors. Effect sizes are interpreted according to Cohen (1988).

Knowles et al. (2015) used loneliness *z*-scores to combine data from the 3-item and 20-item UCLA loneliness scales, which were rated a number of different ways across their studies (in that participants were given a variety of response options). To make our results as comparable as possible to that earlier work, we also calculated *z*-scores. Using *z*-scores also ensured that the Recall scores and Loneliness *z*-scores were on similar scales for the linear mixed model (LMM).

#### Study 1: Social memory task

We first ran a LMM using restricted maximum likelihood (REML) that nested independent variables as repeated measures within each participant. REML is known to be optimal in estimating variance components (Jiang, 2007) and was used because it has several advantages over maximum likelihood (Lin et al., 2013). In the LMM, we included Loneliness *z*-scores, Event Type, Event Valence, Age, the interaction terms between the preceding variables, and Gender, nested within each participant (Gardner et al., 2005). Those models included random intercepts to take into account individual differences between participants, which is equivalent to repeated-measures tests conducted by Gardiner et al. Following Peterson and Brown (2005), we converted the standardized β weights to *r*, providing an effect size that we are able to interpret as weak (*r* ≤ .02), medium (>.02 and <.05), or large (≥. 05) (Cohen, 1988).

Next, we conducted six linear regressions predicting total scores for each of the six categories of event type/valence from Loneliness *z*-scores, with Age included as a continuous covariate. We also examined these relationships in each of the age groups separately.

#### Study 2: ER task

We first sought to replicate the previous results on social versus nonsocial framing of the DANVA-2 task (Knowles et al., 2015) by regressing total DANVA-2 scores on Loneliness *z*-scores and Framing Condition, and the interaction term between the two, using a linear regression model. Because we wanted to know whether age moderated those relationships, Age (as a continuous covariate) and interactions with Age were included in the models. Since both Loneliness and DANVA-2 scores differed between males and females, Gender was also included as a covariate in the models (males had significantly higher loneliness scores than females: *t =* −9.44, *p* < .0001, df *=* 22,052, *R* ^2^ = .004, *d* = .13; males had significantly lower DANVA scores than females *t =* 20.03, *p* < .0001, df *=* 22,052, *R* ^2^ = 0.018, *d* = .28 [see Table 4]).

We also conducted models predicating the total low-intensity scores, following Knowles et al. (2015). We conducted sensitivity analyses excluding DANVA-2 scores under 8 (<3 *SD*s below the mean), due to the negative skew of the data, but the results remained unchanged and we report only the full sample analyses here. In addition, we looked at the low-intensity pictures of each emotion separately (Fear, Sadness, Anger, Happiness) to explore whether the choking under pressure was associated with specific facial emotion cues.

### Results

#### Social memory task

The descriptive statistics for Loneliness and Recall for different event types/valences for this sample can be found in Table 1 (results split by Age Group can be found in Online Supplemental Table S3). Following Gardner et al. (2005), our LMM included Event Type (individual, interpersonal, or collective), Event Valence (positive or negative), Age, Gender, and interactions between all other independent variables except Gender. Results showed a positive association between Loneliness and Recall scores, independent interaction effects between Loneliness × Event Type, Loneliness × Valence, and Valence × Event Type, but no interactions with Age (Table 2). Loneliness was positively associated with Recall for negatively valenced events but not for positive valenced events (Table 2). When the event categories (individual, interpersonal, or collective) were considered separately, loneliness was not significantly associated with Recall performance in any of the models (Online Supplemental Table S4). Age did not show significant main effects in any of the models. Examining each of the six valence/event categories separately, we found no significant effects of Loneliness or the Age × Loneliness interaction on Recall (Online Supplemental Table S5).

**Table 2.** General Linear Model, Using Restricted Maximum Likelihood, Predicting Performance on Memory Task.

	Estimate	95% CI	Converted *r*-value	Degree of freedom	*t*-value	*p*-value
Full model for memory task^e^						
Intercept	0.189	.013 to.365	.239	14,970	2.091	0.037*
Loneliness^a^	0.215	.038 to .391	.265	14,120	2.385	0.017*
Event^b^	0.060	−.138 to .018	.110	13,150	1.494	0.135
Valence^c^	0.096	−.204 to .011	.146	13,150	1.743	0.081
Age	0.002	−.001 to .005	.052	14,120	1.432	0.152
Gender^d^	0.013	−.013 to .038	.063	2,627	1.049	0.294
Loneliness × Event	−0.089	−.169 to .009	.139	13150	−2.159	0.031*
Loneliness × Valence	−0.119	−.229 to .009	.169	13,150	−2.106	0.035*
Event × Valence	−0.067	−.118 to −.016	−.117	13,150	−2.608	0.009**
Loneliness × Age	−0.003	−.007 to .001	−.053	14,120	−1.878	0.060
Event × Age	−0.001	−.003 to .001	−.051	13,150	−1.228	0.219
Valence × Age	−0.001	−.003 to .001	−.051	13,150	−0.842	0.400
Loneliness × Event × Valence	0.050	−.001 to .101	.100	13,150	1.916	0.055
Loneliness × Event × Age	0.001	−.001 to .002	.051	13,150	1.832	0.067
Loneliness × Valence × Age	0.002	−.00004 to −.004	.052	13,150	1.649	0.099
Event × Valence × Age	0.002	−.002 to .002	.052	13,150	0.409	0.682
Loneliness × Event × Valence × Age	0.001	−.002 to .002	.051	13,150	−1.651	0.099
Negatively valenced events only^f^						
Intercept	0.273	−.173 to .373	.323	7,381	5.338	9.69e-08***
Loneliness^a^	0.097	.009 to .185	.147	7,790	2.132	0.033*
Event^b^	−0.006	−.043 to .031	−.056	5,260	−0.333	0.739
Age	0.002	.00004 to .004	.052	7,790	1.793	0.073
Gender^d^	0.021	−.014 to .056	.071	2,627	1.246	0.213
Loneliness × Event	−0.039	−.076 to −.002	−.089	5,260	−2.027	0.043*
Loneliness × Age	−0.001	−.003 to −.001	−.051	7,789	−1.686	0.092
Event × Age	−0.001	−.003 to .001	−.051	5,260	−2.076	0.038*
Loneliness × Event × Age	0.001	−.001 to .003	−.051	5,260	1.686	0.092
Positively valenced events only^g^						
Intercept	0.393	.307 to .472	.442	7,872	9.401	<2e-16***
Loneliness^a^	−0.022	−.085 to .041	−.072	7,324	−0.584	0.559
Event^b^	−0.073	−.106 to −.040	−.123	5,260	−4.334	1.49e-05***
Age	0.001	−.001 to .003	.052	7,324	0.899	0.369
Gender^d^	0.005	−.0185 to .029	.055	2,627	0.423	0.672
Loneliness × Event	0.011	−.022 to .044	.061	5,260	0.637	0.524
Loneliness × Age	0.001	−.001 to .003	.051	7,323	0.426	0.670
Event × Age	−0.001	−.003 to .001	−.051	5,260	−1.706	0.088
Loneliness × Event × Age	−0.001	−.001 to .003	−.051	5,260	−0.617	0.537

*Note*. *N* = 1,291 males and 1,341 females.

^a^ Loneliness *z*-scores, created from the total raw scores on the 4-item UCLA scales, where possible total scores range from 1 to 20; participants are asked to rate how often they felt (i) a lack of companionship, (ii) left out, (iii) isolated from others, and (iv) in tune with others (reverse scored) on a 5-point scale anchored from “never” (1) and to “very often” (5). Higher scores on the 4-item UCLA represented higher loneliness (α = .845). In the social memory task, participants read four short extracts from a diary for an individual of the same sex; after reading the diary, they listed as many events as they could remember from the diary. Two coders, blind to the participants’ loneliness scores, categorized responses as (i) individual events that were positive (e.g., I played a chess match and won), (ii) individual events that were negative (e.g., when cycling to the office, I fell off my bike and hurt my head), (iii) interpersonal events that were positive (e.g., I received a parcel from my cousin (who I am very close to) and it was full of hilarious pictures from our last holiday together), (iv) interpersonal events that were negative (e.g., my best friend let me down: we had made plans to do something at the weekend, but I guess it didn’t matter), (v) collective events that were positive (e.g., my office has got cinema tickets for our good productivity results), and (vi) collective events that were negative (e.g., my group entered a poster competition and we just found out we didn’t win a prize). In the diary, there had been four instances of each kind of event, so there was a maximum of four correct responses for each event. Scores for the number of correctly remembered events were summed for each of the six categories. For analyses, we created categorizes of ^b^Event type (individual, interpersonal or collective) and ^c^Valence (positive or negative); ^d^1 = Male, 2 = Female; ^e^Restricted (residual) maximum likelihood (REML)= 24,198.51; ^f^Restricted (residual) maximum likelihood (REML) = 13673.10; ^g^Restricted (residual) maximum likelihood (REML)= 10,733.30.

*p <.05; **p < .01; ***p < .0001.

Considering the age groups separately, we found a significant positive association between Loneliness and Recall of individual negative events among 25- to 34-year-olds, using our conservative cut off of *p* < .001 (Table 3). The overall effect size was small (*r* = .159, *R* ^2^ = .032). No other significant relationships between loneliness and memory performance were found for the other types of events or in the other age groups at α-level *p* < .001 (Online Supplemental Table S6).

**Table 3.** Linear Model Predicting Memory Task Performance from Loneliness and Gender for Different Kinds of Events in Age Groups for Which Loneliness had a Significant Effect.

	Estimate	95% CI	Converted *r* value	*t*-value	*p*-value
25- to 34-year-olds					
	Negative individual events^a^				
Intercept	0.230	.063 to .397	.28	2.717	0.007**
Loneliness^b^	0.109	.054 to .164	.159	3.921	0.0001 ***
Gender^c^	0.030	−.076 to .136	.080	0.553	0.580

*Note*. *N* = 1,291 males and 1,341 females.

^a^Overall model: F(2,397) = 7.700, *p* = 0.0005.

^b^Loneliness z-scores, created from the total raw scores on the 4-item UCLA scales, where possible total scores range from 1 to 20; participants are asked to rate how often they felt (i) a lack of companionship, (ii) left out, (iii) isolated from others, and (iv) in tune with others (reverse scored) on a 5-point scale anchored from “never” (1) and to “very often” (5). Higher scores on the 4-item UCLA represented higher loneliness (α = .845). In the social memory task, participants read four short extracts from a diary for an individual of the same sex; after reading the diary, they listed as many events as they could remember from the diary. Two coders, blind to the participants’ loneliness scores, categorized responses. Here responses coded as (ii) individual events that were negative (e.g., when cycling to the office, I fell off my bike and hurt my head) were examined. In the diary, there had been four instances of each kind of event, so there was a maximum of four correct responses for individual negative events.

^c^1 = Male, 2 = Female.

** *p* < .01; ***p < .0001.

#### ER Task

Descriptive statistics for Age, Loneliness, and ER performance are given in Table 4, and separately for the different Age Groups, Genders, and Framing Conditions in Online Supplemental Table S7. We first used a linear regression model to look at the scores for the full set of DANVA-2 faces and observed that total scores were greater for females compared to males and those in the nonsocial framing condition compared to those in the social framing condition; higher loneliness was associated with poorer performance, but the effect size was very small (*r* < .04; Table 5). In addition, there was a significant negative relationship between Age and DANVA-2 scores, and, although it did not reach the significance level of *p* < .001, there was also an Age × Loneliness interaction (Table 5). Comparable findings for age and gender were found when considering faces portraying low intensity fear, anger, and sadness (Table 5); for the complete low intensity set, higher loneliness was associated with better ER performance, although, again, the effect size was very small (*r* < .04).

**Table 4.** Descriptive Statistics for Male and Female Participants for Emotion Recognition Task Sample.

		N	Minimum	Maximum	Mean	SD
		Males				
Age		7,159	16.00	99.00	49.29	15.70
Loneliness^a^		7,159	1.00	20.00	10.89	4.58
DANVA-2 scores	Total	7,159	1.00	24.00	17.65	2.96
	Low intensity	7,158	1.00	12.00	7.63	1.78
	Low intensity Fear	6,517	1.00	3.00	1.86	.69
	Low intensity Anger	5,611	1.00	3.00	1.69	.74
	Low intensity Sad	6,995	1.00	3.00	2.38	.70
	Low intensity Happy	7,087	1.00	3.00	2.31	.69
		Females				
Age		14,895	16.00	96.00	49.70	15.23
Loneliness^a^		14,895	1.00	20.00	10.28	4.46
DANVA-2 scores	Total	14,895	2.00	24.00	18.45	2.71
	Low intensity	14,895	1.00	12.00	8.04	1.69
	Low intensity Fear	14,050	1.00	3.00	1.94	.69
	Low intensity Anger	12,157	1.00	3.00	1.75	.75
	Low intensity Sad	14,694	1.00	3.00	2.54	.64
	Low intensity Happy	14,716	1.00	3.00	2.31	.68

*Note*. *N* = sample size; *SD* = standard deviation.

^a^Total raw scores on the 4-item UCLA scale, not z-scores; possible total scores for the total 4-item scale range from 1 to 20; participants are asked to rate how often they felt (i) a lack of companionship, (ii) left out, (iii) isolated from others, and (iv) in tune with others (reverse scored) on a 5-point scale anchored from “never” (1) and to “very often” (5). Higher scores on the 4-item UCLA represented higher loneliness (α = .845). The DANVA-2 includes photos of 24 male and female young adult faces, and those faces were presented individually, for 2 s. Participant were asked to choose which emotion best characterizes the expression of the depicted face (happy, fearful, angry, or sad). There are six faces for each emotion, and three of those are “low intensity,” where the emotion is subtler and, therefore, harder to detect, than in the three “high-intensity” pictures. Of the 13 female faces, 8 show low intensity emotions (two each for all four emotions); 4 of the 11 male faces show low intensity emotion (one each for all four emotions). For the high-intensity emotions, there are two male faces and one female face for each of the emotions except anger, which is the opposite.

**Table 5.** Linear Regression Model Predicting Emotion Recognition (DANVA-2) Scores Across all Ages.

	Coefficients			Variance explained in each step		
	Estimate	95% CI	*R*	*R* ^2^	Δ*R* ^2^	*F* Change
Full DANVA-2 set of faces^d^						
Intercept	18.075	17.662 to 18.488				
Loneliness^a^	−0.137	−.536 to 2.63	.039	.002	.002	33.444, *p* < .001***
Condition^b^	−0.061	−.309 to .187	.045	.002	.001	11.226, *p* < .001***
Age	−0.029	−.039 to −.024	.131	.017	.015	339.277, *p* < .001***
Gender^c^	0.800	.721 to .878	.186	.035	.018	400.606, *p* < .001***
Loneliness × Condition	−0.090	−.343 to .164	.186	.035	.000	1.224, *p* = .269
Loneliness × Age	0.001	−.006 to .009	.187	.035	.000	5.479, *p* = .019*
Condition × Age	0.004	−.001 to .009	.187	.035	.000	2.844, *p* = .092
Loneliness × Condition × Age	0.001	−.004 to .006	.187	.035	.000	.195, *p* = .659
Low intensity set of DANVA-2 faces^e^						
Intercept	7.195	6.939 to 7.451				
Loneliness^a^	0.029	−.219 to .276	.036	.001	.001	28.870, *p* < .001***
Condition^b^	0.008	−.146 to .162	.044	.002	.001	14.417, *p* <.001***
Age	−0.002	−.007 to .003	.045	.002	.000	1.318, *p* = .251
Gender^c^	0.399	.349 to .447	.117	.014	.012	258.632, *p* <.001***
Loneliness × Condition	−0.108	−.265 to .049	.117	.014	.000	.480, *p* = .488
Loneliness × Age	−0.001	−.006 to .004	.118	.014	.000	4.674, *p* = .031*
Condition × Age	0.002	−.001 to .005	.118	.014	.000	1.342, *p* = 2.47
Loneliness × Condition × Age	0.002	−.001 to .005	.118	.014	.000	1.508, *p* = .219
Low intensity set of DANVA-2 faces—Fear^f^						
Intercept	1.569	1.463 to 1.674				
Loneliness^a^	0.074	−.029 to .177	.004	.000	.000	.299, *p* = .584
Condition^b^	0.052	−.011 to .116	.017	.000	.000	5.452, *p* = .020*
Age	0.004	.002 to .005	.061	.003	.003	72.008, *p* <.001***
Gender^c^	0.081	.061 to .101	.082	.003	.003	62.078, *p* < .001***
Loneliness × Condition	−0.041	−.106 to .025	.083	.000	.000	1.695, *p* = .193
Loneliness × Age	−0.001	−.003 to .001	.083	.000	.000	.297, *p* = .586
Condition × Age	−0.001	−.002 to .001	.083	.000	.000	.890, *p* = .346
Loneliness × Condition × Age	0.001	−.001 to .002	.083	.000	.000	.724, *p* = .395
Low intensity set of DANVA-2 faces—Anger^g^						
Intercept	1.521	1.397 to 1.645				
Loneliness^a^	−0.051	−.171 to .070	.021	.000	.000	7.662, *p* = .006**
Condition^b^	−0.010	−.084 to .064	.023	.001	.000	1.677, *p* = .195
Age	0.002	.000 to .004	.060	.004	.003	55.405, *p* <.001***
Gender^c^	0.054	.031 to .078	.060	.005	.001	20.530, *p* <.001***
Loneliness × Condition	0.015	−.061 to .091	.070	.005	.000	3.190, *p* = .074
Loneliness × Age	0.001	−.002 to .003	.071	.005	.000	1.096, *p* = .295
Condition × Age	0.001	−.001 to .002	.071	.005	.000	.460, *p* = .498
Loneliness × Condition × Age	0.001	−.001 to .002	.071	.005	.000	.029, *p* = .866
Low intensity set of DANVA-2 faces—Sadness^h^						
Intercept	2.390	2.291 to 2.488				
Loneliness^a^	−0.009	−.104 to .086	.021	.000	.000	9.467, *p* = .002**
Condition^b^	0.008	−.051 to .067	.024	.001	.001	3.364, *p* = .067
Age	−0.004	−.006 to −.002	.084	.007	.006	140.068, *p* < .001***
Gender^c^	0.154	.136 to .173	.137	.019	.012	262.602, *p* < .001***
Loneliness × Condition	−0.021	−.081 to .039	.138	.019	.000	1.267, *p* = .260
Loneliness × Age	0.001	−.002 to .002	.138	.019	.000	3.006, *p* = .083
Condition × Age	0.001	−.001 to .001	.138	.019	.000	.149, *p* = .699
Loneliness × Condition × Age	0.001	−.001 to .001	.138	.019	.000	.147, *p* = .701
Low intensity set of DANVA-2 faces—Happiness^i^						
Intercept	2.354	2.251 to 2.456				
Loneliness^a^	−0.042	−.141 to .057	.035	.001	.001	27.075, *p* <.001***
Condition^b^	−0.032	−.093 to .029	.035	.001	.000	.213, *p* =.644
Age	−0.001	−.003 to .001	.036	.001	.000	1.176, *p* = .278
Gender^c^	−0.007	−.027 to .012	.036	.001	.000	.518, *p* = .472
Loneliness × Condition	−0.009	−.072 to .054	.036	.001	.000	.000, *p* = .989
Loneliness × Age	0.001	−.002 to .002	.039	.002	.001	4.320, *p* = .038*
Condition × Age	0.001	−.0002 to .002	.040	.002	.000	1.485, *p* = .223
Loneliness × Condition × Age	0.001	−.001 to .001	.040	.002	.000	.115, *p* = .735

*Note*. Estimates are unstandardized β co-efficient.

^a^Loneliness measured using 4-item UCLA loneliness measure transformed into z-scores: participants were asked to rate how often they felt (i) a lack of companionship, (ii) left out, (iii) isolated from others, and (iv) in tune with others (reverse scored) on a 5-point scale anchored from “never” (1) and to “very often” (5). Higher scores on the 4-item UCLA represented higher loneliness (α = .845).

^b^1 = nonsocial framing, 2 = social framing: participants were randomly assigned to either a non-social framing condition (*N* = 11,089) or a social framing condition (*N* = 10,965), and then they completed the emotion recognition task (DANVA-2). The DANVA-2 includes photos of 24 male and female young adult faces, and those faces were presented individually, for 2 s. Participant were asked to choose which emotion best characterizes the expression of the depicted face (happy, fearful, angry, or sad). There are six faces for each emotion, and three of those are “low intensity,” where the emotion is subtler and, therefore, harder to detect, than in the three “high–intensity” pictures. Of the 13 female faces, 8 show low-intensity emotions (two each for all four emotions); 4 of the 11 male faces show low-intensity emotion (one each for all four emotions). For the high-intensity emotions, there are two male faces and one female face for each of the emotions except anger, which is the opposite.

^c^1 = Male, 2 = Female.

^d^ *N* = 7,159 males and 14,895 females, overall model F(8,22045) = 100.30, *p* < 2.2e-16***.

^e^ *N* = 7,159 males and 14,895 females, overall model F(8,22044)= 38.98, *p* < 2.2e-16***.

^f^ *N* = 6,517 males and 14,050 females, overall model F(8,20558)= 17.96, *p* < 2.2e-16***.

^g^ *N* = 5,611 males and 12,157 females, overall model F(8,17759)= 11.27, *p* = 4.787e-16***.

^h^ *N* = 6,995 males and 14,694 females, overall model F(8,21680) = 52.75, *p* < 2.2e-16***.

^i^ *N* = 7,087 males and 14,716 females, overall model F(8,21794) = 4.363, *p* = 2.81e-05***.

* *p* < .05; ***p < .01; **p < .001.

We next explored effects for each age group separately. Table 6 shows those aged 16–24 years, and over 35 years of old performed worse on ER; for those aged 25–34 years, loneliness was associated, instead, with better ER performance. All those effect sizes were very small (*r* < .08). For those aged 25–34 years only, there was also a trend toward a negative interaction effect of Loneliness × Condition on ER performance for low-intensity expressions of DANVA-2 faces (*r* = .18, *p* = .006). That effect appears to have been driven by a choking effect for Fear and Sadness (Online Supplemental Table S9) although effect sizes, again, were small (*rs* = −.122 and −.102 for fear and sadness, respectively). There were no Loneliness × Condition interaction in participants younger than 24 and older than 35 years (total and low-intensity scores: Online Supplemental Table S8; separate emotions Online Supplemental Table S9).

**Table 6.** Linear Regression Models Predicting Total and Low-Intensity-Only Emotion Recognition (DANVA-2) Scores for all Emotions Combined in the Different Age Groups.

Dependent variable		Coefficients			Variance explained in each step		
		Estimate	95% CI	*r*	*R* ^2^	Δ*R* ^2^	*F* Change
16- to 24-year-olds							
	Total DANVA-2 scores^d^						
Intercept		17.126	16.461 to 17.790				
	Loneliness^a^	−0.600	−1.062 to .131	.076	.006	.006	9.008, *p* =.003*
	Framing condition^b^	−0.275	−.561 to .012	.085	.007	.001	2.331, *p* =.127
	Gender^c^	0.973	.689 to 1.266	.184	.034	.027	43.196, *p* <.001***
	Loneliness × Condition	0.247	−.049 to.542	.188	.035	.002	2.685, *p* = .101
	Low-intensity DANVA-2 scores^e^						
Intercept		6.992	6.584 to 7.399				
	Loneliness^a^	−0.232	−.517 to .055	.066	.004	.004	6.884, *p* =.009**
	Framing condition^b^	−0.120	−.296 to .056	.072	.005	.001	1.370, *p* = .242
	Gender^c^	0.513	−.334 to .693	.158	.025	.020	31.716, *p* <.001***
	Loneliness × Condition	0.073	−.108 to .254	.159	.025	.000	.629, *p* = .428
25- to 34-year-olds							
	Total DANVA-2 scores^f^						
Intercept		16.820	16.358 to 17.283				
	Loneliness^a^	0.282	−.037 to .599	.057	.003	.003	9.266, *p* = .002**
	Framing condition^b^	0.096	−.099 to .291	.059	.003	.000	.691, *p* = .406
	Gender^c^	1.026	−.819	.188	.035	.032	94.056, *p* < .001***
	Loneliness × Condition	−0.257	−.459 to .054	.193	.035	.002	6.183, *p* = .013*
	Low-intensity DANVA-2 scores^g^						
Intercept		6.740	6.44 to 7.036				
	Loneliness^a^	0.247	.043 to .450	.032	.001	.001	2.998, *p* = .083
	Framing condition^b^	0.070	−.055 to .194	.037	.001	.001	.894, *p* = .344
	Gender^c^	0.621	.488 to .754	.173	.030	.029	83.904, *p* <.001***
	Loneliness × Condition	−0.181	.006	.180	.032	.003	7.464, *p* = .006**
35- to 64-year-olds							
	Total DANVA-2 score^h^						
Intercept		16.755	16.530 to 16.980				
	Loneliness^a^	−0.058	−.203 to .087	.048	.002	.002	31.527, *p* <.001***
	Framing condition^b^	0.2073	.114 to .300	.059	.004	.001	16.766, *p* < .001
	Gender^c^	0.725	.625 to .825	.134	.018	.014	201.424, *p* < .001***
	Loneliness × Condition	−0.036	−.128 to .056	.134	.018	.000	.586, *p* = .444
	Low-intensity DANVA-2 scores^i^						
Intercept		7.203	7.065 to 7.341				
	Loneliness^a^	−0.047	−.136 to .042	.037	.001	.001	18.298, *p* <.001***
	Framing conditionb	0.126	.068 to .163	.051	.003	.001	16.827, *p* < .001
	Gender^c^	0.356	.295 to .418	.109	.012	.009	128.735, *p* <.001***
	Loneliness × Condition	−0.003	−.060 to .053	.109	.012	.000	.013, *p* = .909
65+ years-old							
	Total DANVA-2 scores^j^						
Intercept		16.001	15.578 to 16.424				
	Loneliness^a^	−0.104	−.386 to .179	.050	.003	.003	9.973, *p* = .002**
	Framing condition^b^	0.096	−.083 to .275	.053	.003	.000	.993, *p* = .319
	Gender^c^	0.783	.596 to .970	.140	.020	.017	67.616, *p* <.001***
	Loneliness × Condition	−0.017	−.195 to .162	.140	.020	.000	.034, *p* = .855
	Low-intensity DANVA-2 scores^k^						
Intercept		7.115	6.853 to 7.377				
	Loneliness^a^	−0.078	−.253 to .097	.033	.001	.001	4.387, *p* = .036*
	Framing condition^b^	0.089	−.022 to .199	.041	.002	.001	2.125, *p* = .145
	Gender^c^	0.313	.197 to .429	.094	.009	.007	28.242, *p* <.001***
	Loneliness × Condition	0.017	−.93 to .128	.094	.009	.000	.095, *p* = .759

*Note*. Estimates are unstandardized β co-efficient.

^a^Loneliness measured using 4-item UCLA loneliness measure transformed into z-scores: participants were asked to rate how often they felt (i) a lack of companionship, (ii) left out, (iii) isolated from others, and (iv) in tune with others (reverse scored) on a 5-point scale anchored from “never” (1) and to “very often” (5). Higher scores on the 4-item UCLA represented higher loneliness (α = .845).

^b^1 = nonsocial framing, 2 = social framing: participants were randomly assigned to either a non-social framing condition (*N* = 11,089) or a social framing condition (*N* = 10,965), and then they completed the emotion recognition task (DANVA-2). The DANVA-2 includes photos of 24 male and female young adult faces, and those faces were presented individually, for 2 s. Participant were asked to choose which emotion best characterizes the expression of the depicted face (happy, fearful, angry, or sad). There are six faces for each emotion, and three of those are “low intensity,” where the emotion is subtler and, therefore, harder to detect, than in the three “high–intensity” pictures. Of the 13 female faces, 8 show low-intensity emotions (two each for all four emotions); 4 of the 11 male faces show low-intensity emotion (one each for all four emotions). For the high-intensity emotions, there are two male faces and one female face for each of the emotions except anger, which is the opposite.

^c^1 = Male, 2 = Female.

^d^ *N* = 555 males and 1,015 females, overall model: F(4,1565) = 14.40, *p* = 1.505e-11***.

^e^ *N* = 555 males and 1,015 females, overall model: F(4,1564) = 10.19, *p* = 3.829e-08***.

^f^ *N* = 954 males and 1,905 females, overall model: F(4,2854) = 27.68, *p* ≤ 2.2e-16***.

^g^ *N* = 954 males and 1,905 females, overall model: F(4,2854) = 23.89, *p* ≤ 2.2e-16***.

^h^ *N* = 4,368 males and 9,325 females, overall model: F(4,13688) =62.76, *p* ≤ 2.2e-16***.

^i^ *N* = 4,368 males and 9,325 females, overall model: F(4,13688) = 41.05, *p* ≤ 2.2e-16***.

^j^ *N* = 1,282 males and 2,650 females, overall model: F(4,3927) = 19.70, *p* = 4.572e-16***.

^k^ *N* = 1,282 males and 2,650 females, overall model: F(4,3927) = 8.72, *p* = 5.248e-07***.

* *p* < .05; ***p < .01; **p < .001.

## Discussion

In the current study, we set out to understand whether those who reported higher loneliness, regardless of age, (1) remembered more socially relevant information than their non-lonely peers because they desired social inclusion and (2) were impaired from reconnection because they were anxious to do well under social situations. In previous work, using an incidental social memory task, undergraduates reporting loneliness were found to have better recall of both positive and negative social information (Gardner et al., 2005). In addition, undergraduates who reported loneliness only showed impairments in ER, a key ability in social interaction, when the task was framed as a social task (Gardner et al., 2005; Knowles et al., 2015). We wanted to determine whether those patterns of results could be replicated in a larger sample of young adults and were consistent across people of different ages who reported loneliness. Contrary to our expectations, we found effects only for those participants aged 25–34 years, where (a) loneliness was positively associated with recall of negative individual (non-social) events, and (b) those who reported loneliness appeared to choke under social pressure, performing worse in the social framing task, that looked to be specifically related to the identification of fear and sadness, although those differences did not reach our conservative cutoff of *p* < .001. The small effect sizes suggest a need to be cautious (Sullivan & Feinn, 2012), but are perhaps expected here compared to laboratory settings where other influences can be controlled. Thus, while the current design reduces the likelihood of laboratory artefacts, such as demand characteristics, and, thus, provides better estimates of the effect sizes we might expect to observe in real-world contexts, the diversity of our sample (nationality, age, SES, employment status, education level, visual impairment) and the noise that accompanies such dissimilarity, even small effect sizes may be meaningful and might be larger under more controlled conditions.

Contrary to previous research showing that undergraduates reporting loneliness were attuned to both positive and negative social information, we found no such effects: In our sample, loneliness was not associated with increased social monitoring in any age group. Instead, we found that, for participants aged 25–34 years, loneliness was associated with recall of negative individual information, suggesting that loneliness may prime *individuals* in this age group for negative nonsocial information. While that effect was small, it might be explained by an adaptation of the predator evasion defense, previously documented in socially isolated rodents and applied to humans (Cacioppo & Cacioppo, 2014). According to that evolutionary model of loneliness, loneliness increases the motivation for short-term self-preservation, which means that, along with increased social monitoring we would also expect to see increases in self-focused behavior (Cacioppo et al., 2006), that reflect concern for self-interest and self-welfare. Why we might see that effect for participants aged 25–34 years in our sample is uncertain, but the findings raise the question of whether the mechanism that promotes belongingness and includes social monitoring and self-preservation changes focus across development, or may work differently between individuals, being driven perhaps by the temporal nature of the loneliness experience. It is striking that we also found some evidence for ‘choking under social pressure’ in this same age group (and only there): Underperforming in social situations might be related to this greater sensitivity to negative information regarding the self.

We did not find evidence that loneliness enhanced social monitoring in the BBC Loneliness Experiment sample and that may reflect the fact that loneliness experiences motivate people differently: For some, loneliness may motivate them to attend to social opportunities to ensure reconnection; for others, loneliness may increase the motivation for short-term self-preservation, leading to self-focused, and avoidant, behavior. Such differences might reflect the temporality of loneliness. It is possible that transitory loneliness is associated with increased social monitoring, but chronic, prolonged loneliness, for some individuals, is associated with a focus on self-preservation at the expense of re-affiliation. For other individuals, prolonged loneliness might be associated with heightened sensitivity to negative emotions linked to potential rejection, as proposed elsewhere (Qualter, Vanhlast, et al., 2015).

It has been argued that hypersensitivity to negative social information could lead to social avoidance and increased loneliness (Cacioppo & Hawkley, 2009; Gardner et al., 2005; Qualter, Vanhalst, et al., 2015), which may represent a flawed self-preservation strategy (McQuaid et al., 2014). Exploration of loneliness subgroups and how temporal experiences of loneliness motivate reconnection or social avoidance is important. The fact is that, for some people, the emotional distress of loneliness, and the acute sensitivity to negative social stimuli that accompanies it, may increase an individual’s motivation to self-preservation over time, promoting social avoidance, and potential further problems, such as depression, anxiety, and prolonged loneliness; individuals may formulate social goals aimed at avoiding social situations due to the risk of adverse social judgments. The next step in research, then, should be to explore individual differences in motivation and social goals to determine how those influence social monitoring and choking under pressure across development.

Knowles et al. (2015) found that undergraduate students reporting loneliness performed worse than their peers when they thought their social skills were being tested, but they did just as well as their peers on the same test when they thought the task measured cognitive skills. In the current study, we found some evidence that individuals aged 25–34 years (i.e., slightly older than participants in the Knowles et al., 2015 study) might choke under pressure; we did not find that any trends toward that effect in any other age group. Thus, choking under social pressure appeared, in the current sample, to be restricted to early adulthood. It is possible that this age group is the most susceptible to choking under pressure because they are trying to establish new relationships and are more likely to be adjusting to new social roles in work and at home, having new social interactions more frequently than other groups. Others, such as Knowles et al. (2015), have speculated that those over 65 years would be unlikely to choke under pressure because their social interactions occur within established relationships. We supported that idea, but we found that might also be the case for aged 35–64 years as, after all, social networks in middle age are likely to be strongly determined by work contexts and family ties, which tend to be relatively stable.

Our findings support those from the earlier work (Knowles’ et al., 2015) that people reporting loneliness did not suffer ER deficits. Indeed, individuals reporting loneliness in the current study performed the task just as well as their peers who did not report loneliness. However, there was some indication in the data reported here (though not at our conservative *p* < .001 level) that for those aged 16–24 years, loneliness was associated with worse ER; that was not the case for other age groups. The finding that adolescents reporting loneliness have problems with ER has been reported elsewhere (Wols et al., 2015), but those authors argue that other emotion related skills should also be investigated to fully explore how loneliness is related to understanding emotion knowledge in social contexts. Thus, future work is needed to establish whether loneliness influences how we understand our own and other people’s emotions in social encounters.

The DANVA-2 task is limited because it only measures whether people can recognize emotions in the faces of others, rather than also taking into account body language, for example. Moreover, the task does not provide information about whether a person feels confident enough to translate those ER skills into social contexts (Qualter, Dacre-Pool, et al., 2015). Given previous research demonstrating that distinct self-efficacy beliefs play a role in managing negative and positive affect (Caprara, Fida et al., 2008; Caprara, Giunta et al., 2008), future work should examine whether loneliness is associated prospectively with people’s beliefs about whether they can successfully perceive, use, and understand emotional information. It is possible that loneliness does not affect ER accuracy, but it may not reduce self-confidence in ER, which could contribute to prolonged experiences of loneliness.

### Strengths, Limitations, and Future Studies

While previous work (Knowles et al., 2015) has pointed out the need to examine whether heightened social monitoring and “choking under pressure” are associated with loneliness across ontogeny, the current study is the first to do that. It is a strength of the study that we have a large number of participants from many countries across the world who completed the experimental tasks. However, the current study does not include a representative sample, which means self-selection bias may be present, especially because those that completed the survey are likely to have had a particular interest in understanding loneliness and/or improving that experience for themselves or others. In addition, most participants came from mainly Western countries, and future research will want to explore the associations in other cultures more thoroughly. Further, the BBC Loneliness Experiment was only open to people over the age of 16 years, which means our findings are limited to understanding social monitoring of those older than that. While there is some empirical work with children and adolescents that explores loneliness and social monitoring (see Qualter, Vanhlast et al., 2015, for details), future work will want to extend that work to fully understand the association across ontogeny.

The fact that we did not include a mood measure in the current study could be viewed as a weakness. It had been used in the work we attempted to replicate, but because mood did not significantly interact with loneliness to predict recall of social information or ER in those studies, mood was not included in the current study.

Despite the small effect sizes, the current findings may have real practical significance: Although the influence on ER and recall of information may be small, it may lead to social avoidance and increased loneliness. Thus, future work will want to explore the prospective relations between social monitoring, choking under pressure, social avoidance, and loneliness and to continue to explore age differences in those over-time processes. Through such future work, we will learn whether targeting social monitoring as part of an intervention could help maintain or worsen loneliness across ontogeny.

Another important consideration for future work is the need to explore how transitory and prolonged loneliness are related to social monitoring profiles. It is possible that prolonged loneliness is associated with specific patterns, while transitory loneliness is association with different ones. Such differences may explain the inconsistent findings presented in the introduction, with our findings adding to that complexity. It may be the case that those who progress from transitory to chronic loneliness are those who engage in social avoidance as a response to heightened social sensitivity or a self-preservation goal at the expense of re-affiliation. Future work will want to examine avoidance motivation as a potential underlying process that determines the type of social monitoring the individual engages in. Such work would need to examine whether individuals whose sense of self is contingent on the judgments and approval of peers are more likely to be motivated to avoid interaction when they experience loneliness. If so, they might be more prone to socially monitor for threats and, thus, prolonged loneliness because they exhibit critical appraisals, helplessness, and anxiety (Slavich et al., 2009; Slavich et al., 2010).

## Conclusions

The present study makes a novel contribution to understanding how loneliness is related to ER accuracy and recall of positive and negative social information. We do that by examining data from the large sample of participants who completed different cognitive tasks as part of the BBC Loneliness Experiment. We also investigate whether the associations are found across different age groups. We found specific patterns of social monitoring only among participants aged 25–34 years, with lonely participants recalling more negative information, and ‘choking under pressure’ during an ER task that was framed as diagnostic of social performance. We argue that those reporting loneliness in this age group self-monitor too much, as evident by their increased recall of negative personal information and their “choking under pressure” during the ER task because they are distracted. Thus, it could be that different psychological processes underlie loneliness at different life stages or are related over time to different types of loneliness. Overall, this article highlights the importance of taking a life course approach to studying the potential cognitive biases in loneliness.

## Supplemental material

IJBD_Supplementary_Materials_Resub_FINAL_V2 - Choking under pressure: Does it get easier with age? How loneliness affects social monitoring across the life span Click here for additional data file. IJBD_Supplementary_Materials_Resub_FINAL_V2 for Choking under pressure: Does it get easier with age? How loneliness affects social monitoring across the life span by Ellie Pearce, Manuela Barreto, Christina Victor, Claudia Hammond, Alice M. Eccles, Matthew T. Richins, Alisha O’Neil, Megan L. Knowles and Pamela Qualter in International Journal of Behavioral Development
