# Supplementary material for: Choking under pressure: Does it get easier with age? How loneliness affects social monitoring across the life span
Source: Int J Behav Dev. 2020 Dec 20;46(1):50–62. doi: 10.1177/0165025420979369 (PMC8727830; doi:10.1177/0165025420979369)
Supplement: IJBD_Supplementary_Materials_Resub_FINAL_V2 - Choking under pressure: Does it get easier with age? How loneliness affects social monitoring across the life span [file IJBD_Supplementary_Materials_Resub_FINAL_V2.pdf]

Supplementary Material for Paper Entitled 'Choking Under Pressure: Does it get easier with age? How Loneliness relates to Social Monitoring across the Lifespan'.

DANVA-2 task

Table S1: Frequency of respondents living in different countries at the time of data collection.

Country	Frequency of respondents	Percentage of sample
Afghanistan	8	.0
Albania	4	.0
Antarctica	1	.0
Algeria	2	.0
Andorra	3	.0
Antigua and Barbuda	1	.0
Azerbaijan	1	.0
Argentina	21	.1
Australia	475	2.2
Austria	33	.1
Bahamas, The	1	.0
Bahrain	6	.0
Bangladesh	13	.1
Armenia	1	.0
Barbados	8	.0
Belgium	62	.3
Bermuda	6	.0
Bhutan	1	.0
Bolivia	1	.0
Bosnia and Herzegovina	3	.0
Botswana	1	.0
Brazil	31	.1
Belize	2	.0
Solomon Islands	1	.0
British Virgin Islands	2	.0
Brunei	5	.0
Bulgaria	20	.1
Burma	3	.0
Belarus	3	.0

Cambodia	4	.0
Cameroon	2	.0
Canada	529	2.4
Cayman Islands	1	.0
Sri Lanka	2	.0
Chile	22	.1
China	43	.2
Taiwan	17	.1
Colombia	12	.1
Costa Rica	9	.0
Croatia	10	.0
Czech Republic	24	.1
Denmark	39	.2
Dominica	1	.0
Ecuador	7	.0
El Salvador	1	.0
Ethiopia	1	.0
Estonia	6	.0
Faroe Islands	2	.0
Fiji	1	.0
Finland	30	.1
France	297	1.3
French Polynesia	1	.0
Gambia, The	2	.0
Occupied Palestinian Territories	1	.0
Germany	269	1.2
Ghana	8	.0
Gibraltar	4	.0
Greece	42	.2
Grenada	1	.0
Guadeloupe	1	.0
Guatemala	3	.0
Guyana	2	.0
Honduras	1	.0
Hong Kong	45	.2
Hungary	16	.1
Iceland	8	.0
India	121	.5
Indonesia	32	.1
Iran	3	.0
Iraq	3	.0

Ireland	229	1.0
Israel	18	.1
Italy	104	.5
Jamaica	9	.0
Japan	51	.2
Kazakhstan	4	.0
Jordan	10	.0
Kenya	26	.1
Korea (South)	12	.1
Kuwait	7	.0
Laos	1	.0
Lebanon	9	.0
Lesotho	1	.0
Latvia	5	.0
Liberia	1	.0
Lithuania	10	.0
Luxembourg	12	.1
Macao	1	.0
Madagascar	1	.0
Malawi	4	.0
Malaysia	44	.2
Maldives	2	.0
Malta	18	.1
Martinique	1	.0
Mauritius	8	.0
Mexico	30	.1
Monaco	1	.0
Mongolia	1	.0
Moldova	1	.0
Montenegro	1	.0
Morocco	1	.0
Mozambique	1	.0
Oman	3	.0
Namibia	1	.0
Nepal	6	.0
Netherlands	116	.5
New Zealand	151	.7
Norway	63	.3
Pakistan	20	.1
Panama	7	.0
Paraguay	1	.0

Peru	6	.0
Philippines	29	.1
Poland	48	.2
Portugal	39	.2
Puerto Rico	2	.0
Qatar	14	.1
Russia	67	.3
St Kitts and Nevis	1	.0
St Lucia	3	.0
St Vincent and the Grenadines	3	.0
Saudi Arabia	16	.1
Senegal	1	.0
Serbia	10	.0
Sierra Leone	3	.0
Singapore	82	.4
Slovakia	8	.0
Vietnam	10	.0
Slovenia	7	.0
South Africa	84	.4
Zimbabwe	4	.0
Sweden	77	.3
Switzerland	100	.5
Thailand	39	.2
Tonga	1	.0
Trinidad and Tobago	19	.1
United Arab Emirates	41	.2
Tunisia	2	.0
Turkey	72	.3
Uganda	12	.1
Ukraine	10	.0
Macedonia	5	.0
Egypt	17	.1
Guernsey	16	.1
Jersey	11	.0
Isle Of Man	23	.1
Tanzania	7	.0
United States	1883	8.5
Uruguay	3	.0
Venezuela	1	.0
Samoa	1	.0
Yemen	2	.0

Zambia	13	.1
Cyprus (European Union)	8	.0
Cyprus (Non-European Union)	2	.0
Spain and Canary Islands	140	.6
England	9367	42.5
Northern Ireland	181	.8
Scotland	1078	4.9
Wales	527	2.4
Great Britain	4376	19.8
Kosovo	2	.0
Not otherwise Specified	66	.3
Missing data	181	.8

Table S2: Details of missing data for the two studies

Study	Reason for missing data	Number of participants excluded	Remaining sample size
Study 1: social memory task			9273 completed the memory task
	Only subsample coded[a]	6502	2771
	Missing age data	73	2698
	Missing gender information or did not identify as male or female	8	2690
	Missing loneliness scores	53	2637
	Missing memory scores	5	2632
Study 2: emotion recognition			55060 completed the survey
	Aged under 16 years	86	54974
	Missing age data (measured continuously in years)	7593	47381
	Missing gender information	9	47372
	Did not identify as male or female (i.e. as 'Other' or 'Prefer not to say')[b]	468	46904
	Missing loneliness scores	1450	45454
	Missing DANVA-2 scores	23,400	22054
	Presentation error[c]	0	22054

[a] We tried to ensure 200 participants in all unmerged age categories (16-24 years, 25-34 years, 35-44 years, 45-54 years, 55-64 years, 65-74 years, 75+ years) for both males and females. Once participants with missing data were removed, there were at least 190 participants in each age/gender category except for the 75+years category, which had 96 males and 163 females.

[b] Excluded because those sample sizes were substantially smaller than the male (N=15,221) and female (N=31,683) sample sizes.

Table S3: Descriptive statistics for loneliness and memory scores for different event types in the different age groups and genders

Age Group	Gender		N	Minimum	Maximum	Mean	Standard Deviation
16-24	Male	Loneliness[a]	190	-1.47	2.01	0.31	0.95
		Individual Positive[b]	190	.00	2.00	.34	.48
		Individual Negative[b]	190	.00	3.00	.25	.51
		Interpersonal Positive[b]	190	.00	3.00	.27	.55
		Interpersonal Negative[b]	190	.00	3.00	.34	.60
		Collective Positive[b]	190	.00	2.00	.14	.40
		Collective Negative[b]	190	.00	3.00	.19	.51
	Female	Loneliness[a]	198	-1.47	2.01	0.21	0.94
		Individual Positive[b]	198	.00	2.00	.27	.49
		Individual Negative[b]	198	.00	3.00	.30	.58
		Interpersonal Positive[b]	198	.00	3.00	.23	.53
		Interpersonal Negative[b]	198	.00	4.00	.40	.70
		Collective Positive[b]	198	.00	2.00	.16	.40
		Collective Negative[b]	198	.00	3.00	.28	.57
25-34	Male	Loneliness[a]	203	-1.47	2.01	0.11	0.96
		Individual Positive[b]	203	.00	2.00	.33	.49
		Individual Negative[b]	203	.00	3.00	.2709	.55
		Interpersonal Positive[b]	203	.00	2.00	.16	.44
		Interpersonal Negative[b]	203	.00	4.00	.37	.74
		Collective Positive[b]	203	.00	3.00	.20	.48
		Collective Negative[b]	203	.00	3.00	.21	.49
	Female	Loneliness[a]	197	-1.47	2.01	-0.09	0.96
		Individual Positive[b]	197	.00	2.00	.31	.49
		Individual Negative[b]	197	.00	3.00	.28	.54
		Interpersonal Positive[b]	197	.00	3.00	.24	.56
		Interpersonal Negative[b]	197	.00	2.00	.35	.58
		Collective Positive[b]	197	.00	3.00	.17	.50
		Collective Negative[b]	197	.00	3.00	.24	.55
35-64	Male	Loneliness[a]	605	-1.47	2.01	0.17	1.01
		Individual Positive[b]	605	.00	3.00	.39	.57
		Individual Negative[b]	605	.00	3.00	.34	.62
		Interpersonal Positive[b]	605	.00	3.00	.21	.52
		Interpersonal Negative[b]	605	.00	4.00	.33	.68

		Collective Positive[b]	605	.00	3.00	.16	.44
		Collective Negative[b]	605	.00	4.00	.24	.57
	Female	Loneliness[a]	591	-1.47	2.01	-0.02	1.00
		Individual Positive[b]	591	.00	3.00	.35	.52
		Individual Negative[b]	591	.00	4.00	.35	.65
		Interpersonal Positive[b]	591	.00	3.00	.25	.51
		Interpersonal Negative[b]	591	.00	4.00	.32	.60
		Collective Positive[b]	591	.00	3.00	.14	.41
		Collective Negative[b]	591	.00	3.00	.25	.56
65+	Male	Loneliness[a]	293	-1.47	2.01	-.27	0.96
		Individual Positive[b]	293	.00	2.00	.30	.48
		Individual Negative[b]	293	.00	3.00	.33	.56
		Interpersonal Positive[b]	293	.00	2.00	.10	.31
		Interpersonal Negative[b]	293	.00	3.00	.27	.60
		Collective Positive[b]	293	.00	3.00	.12	.40
		Collective Negative[b]	293	.00	3.00	.18	.47
	Female	Loneliness[a]	293	-1.47	2.01	-0.31	0.97
		Individual Positive[b]	355	4.00	20.00	9.34	4.46
		Individual Negative[b]	355	.00	2.00	.36	.50
		Interpersonal Positive[b]	355	.00	3.00	.34	.61
		Interpersonal Negative[b]	355	.00	3.00	.20	.47
		Collective Positive[b]	355	.00	3.00	.32	.65
		Collective Negative[b]	355	.00	2.00	.12	.36
		Loneliness[a]	355	.00	4.00	.23	.57

[a] z-scores of total scores on the four-item UCLA scale.
[b] Memory recall scores for different types of event: individual (positive or negative valence), interpersonal (positive or negative valence), collective (positive or negative valence).

Table S4: Hierarchical linear model predicting performance on memory task for different types of event (individual, interpersonal or collective).

	Estimate	SE	Degree of freedom	t-value	p-value
Individual events[d]					
Intercept	0.194	0.069	3971	2.804	0.005 **
Loneliness[a]	0.120	0.067	3288	1.796	0.073
Valence [b]	0.060	0.040	2628	1.472	0.141
Age	0.002	0.001	3287	1.620	0.105
Gender[c]	-0.001	0.016	2627	-0.044	0.965
Loneliness *Valence	-0.065	0.042	2628	-1.551	0.121
Loneliness *Age	-0.002	0.001	3287	-1.617	0.106
Valence*Age	-0.001	0.001	2628	-0.878	0.380
Loneliness *Valence *Age	0.001	0.001	2628	1.350	0.177
Interpersonal events[e]					
Intercept	0.435	0.065	4631	6.663	2.99e-11 ***
Loneliness[a]	0.050	0.061	3707	0.822	0.411
Valence [b]	-0.098	0.036	2628	-2.731	0.006 **
Age	-0.001	0.001	3707	-0.499	0.618
Gender[c]	0.031	0.018	2617	1.691	0.091
Loneliness *Valence	-0.026	0.003	2628	-0.720	0.471
Loneliness *Age	0.001	0.001	3707	0.148	0.882
Valence *Age	-0.001	0.001	2628	-0.714	0.475
Loneliness *Valence *Age	-0.001	0.001	2628	-0.171	0.864
Collective events[f]					
Intercept	0.299	0.056	4567	5.350	9.24e-08 ***
Loneliness[a]	-0.058	0.052	3659	-1.113	0.266
Valence [c]	-0.073	0.031	2628	-2.348	0.019 *
Age	0.001	0.001	3659	0.116	0.908
Gender[d]	0.010	0.015	2627	0.637	0.524

Loneliness *Valence	0.035	0.032	2628	1.109	0.268
Loneliness *Age	0.001	0.001	3659	0.831	0.406
Valence *Age	-0.001	0.001	2628	-0.482	0.630
Loneliness *Valence *Age	-0.001	0.001	2628	-0.937	0.349

*p<0.05, **p<0.01, ***p<0.0001

[a] Loneliness z-scores

[b] Valence: positive or negative

[c] 1=Male, 2=Female

[d] Restricted (residual) maximum likelihood (REML)= 8859.70

[e] Restricted (residual) maximum likelihood (REML)= 888.40

[f] Restricted (residual) maximum likelihood (REML)= 7217.20

Table S5: Linear models predicting recall of the six valence/event categories across the whole sample

	Estimate	SE	t-value	p-value
Positive individual events[c]				
Intercept	0.337	.041	8.263	2.23e-16 ***
Loneliness[a]	-0.011	0.028	-0.375	0.708
Age	0.001	0.001	1.234	0.217
Gender[b]	-0.016	0.020	-0.777	0.437
Loneliness *Age	0.001	0.001	0.184	0.854
Negative individual events[d]				
Intercept	0.232	0.047	4.941	8.27e-07 ***
Loneliness[a]	0.055	0.033	1.691	0.091
Age	0.001	0.001	2.157	0.031 *
Gender[b]	0.014	0.023	0.614	0.539
Loneliness *Age	-0.001	0.001	-1.568	0.117
Positive interpersonal events[e]				
Intercept	0.216	0.039	5.550	3.15e-08 ***
Loneliness[a]	-0.002	0.027	-0.089	0.929
Age	-0.002	0.001	-3.079	0.002 **
Gender[b]	0.046	0.019	2.380	0.017 *
Loneliness *Age	-0.001	0.001	-0.134	0.894
Negative interpersonal events[f]				
Intercept	0.359	0.051	7.074	1.92e-12 ***
Loneliness[a]	0.029	0.035	0.647	0.517
Age	-0.001	0.001	-1.587	0.113
Gender[b]	0.016	0.025	0.622	0.534
Loneliness *Age	0.001	0.001	0.079	0.937
Positive collective events[g]				
Intercept	0.189	0.033	5.695	1.37e-08 ***
Loneliness[a]	0.011	0.023	0.488	0.626
Age	-0.001	0.001	-1.023	0.306
Gender[b]	-0.015	0.016	-0.884	0.377

Loneliness *Age	-0.001	0.001	-0.691	0.490
Negative collective events[h]				
Intercept	0.190	0.043	4.427	9.94e-06 ***
Loneliness[a]	-0.022	0.030	-0.735	0.462
Age	-0.001	0.001	-0.340	0.734
Gender[b]	0.034	0.021	1.600	0.110
Loneliness *Age	0.001	0.001	0.454	0.650

*p<0.05, **p<0.01, ***p<0.0001

[a] Loneliness z-scores

[b] 1=Male, 2=Female

[c] Overall model: $F(4,2627)$= 0.653, p = 0.625, *adjusted* R^2=-0.001

[d] Overall model: $F(4,2627)$= 2.010, p = 0.091, *adjusted* R^2=0.002

[e] Overall model: $F(4,2627)$= 3.709, p = 0.005, *adjusted* R^2=0.004

[f] Overall model: $F(4,2627)$= 1.994, p = 0.093, *adjusted* R^2=0.002

[g] Overall model: $F(4,2627)$= 0.580, p = 0.678, *adjusted* R^2=-0.001

[h] Overall model: $F(4,2627)$= 0.938, p = 0.440, *adjusted* R^2=-0.001

Table S6: Linear models predicting memory task performance from loneliness and gender for different kinds of events in all the different age groups

	Estimate	SE	t-value	p-value
16-24 year olds				
Positive individual events[c]				
Intercept	0.393	0.080	4.940	1.17e-06 ***
Loneliness[a]	0.019	0.026	0.723	0.470
Gender[b]	-0.062	0.050	-1.252	0.211
Negative individual events[d]				
Intercept	0.201	0.089	2.250	0.025 *
Loneliness[a]	0.016	0.029	0.528	0.598
Gender[b]	0.047	0.056	0.844	0.399
Positive interpersonal events[e]				
Intercept	0.300	0.088	3.400	7.44e-04 ***
Loneliness[a]	0.012	0.029	0.375	0.708
Gender[b]	-0.035	0.055	-0.635	0.526
Negative interpersonal events[f]				
Intercept	0.246	0.107	2.307	0.022 *
Loneliness[a]	0.069	0.035	1.960	0.051
Gender[b]	0.069	0.066	1.044	0.297
Positive collective events[g]				
Intercept	0.115	0.066	1.749	0.081
Loneliness[a]	0.005	0.022	0.228	0.819
Gender[b]	0.020	0.041	0.494	0.622
Negative collective events[h]				
Intercept	0.111	0.089	1.253	0.211
Loneliness[a]	0.002	0.030	0.057	0.955
Gender[b]	0.083	0.055	1.507	0.133
25-34 year olds				
Positive individual events[i]				
Intercept	0.353	0.077	4.556	6.96e-06 ***
Loneliness[a]	-0.025	0.025	-0.969	0.333

Gender[b]	-0.020	0.049	-0.410	0.682
Negative individual events [j]				
Intercept	0.230	0.085	2.717	0.007**
Loneliness[a]	0.109	0.028	3.921	0.0001 ***
Gender[b]	0.030	0.054	0.553	0.580
Positive interpersonal events[k]				
Intercept	0.078	0.080	0.978	0.329
Loneliness[a]	0.028	0.026	1.059	0.290
Gender[b]	0.082	0.051	1.606	0.109
Negative interpersonal events[l]				
Intercept	0.382	0.105	3.623	0.0003 ***
Loneliness[a]	0.023	0.035	0.676	0.500
Gender[b]	-0.015	0.067	-0.218	0.827
Positive collective events[m]				
Intercept	0.212	0.077	2.754	0.006**
Loneliness[a]	0.032	0.025	1.255	0.210
Gender[b]	-0.018	0.049	-0.372	0.710
Negative collective events[n]				
Intercept	0.186	0.083	2.257	0.025 *
Loneliness[a]	-0.021	0.027	-0.769	0.4423
Gender[b]	0.028	0.052	0.529	0.597
	35-64 year olds			
Positive individual events[o]				
Intercept	0.438	0.050	8.759	<2e-16 ***
Loneliness[a]	-0.023	0.016	-1.449	0.148
Gender[b]	-0.042	0.032	-1.335	0.182
Negative individual events[p]				
Intercept	0.338	0.058	5.840	6.73e-09 ***
Loneliness[a]	-0.016	0.018	-0.891	0.373
Gender[b]	0.005	0.037	0.139	0.889
Positive interpersonal events [q]				
Intercept	0.184	0.047	3.897	0.0001***
Loneliness[a]	-0.027	0.015	-1.847	0.065
Gender[b]	0.032	0.030	1.075	0.283
Negative interpersonal events[r]				
Intercept	0.351	0.059	5.955	3.41e-09 ***
Loneliness[a]	-0.004	0.018	-0.191	0.849
Gender[b]	-0.016	0.037	-0.440	0.660

Positive collective events[s]					
Intercept		0.194	0.039	4.950	8.5e-07 ***
	Loneliness[a]	-0.025	0.012	-2.002	0.046 *
	Gender[b]	-0.029	0.025	-1.188	0.235
Negative collective events[t]					
Intercept		0.230	0.052	4.441	9.78e-06 ***
	Loneliness[a]	-0.021	0.016	-1.295	0.196
	Gender[b]	0.010	0.033	0.312	0.755
65 years and over: Negative interpersonal events[e]					
Positive individual events[u]					
Intercept		0.229	0.063	3.615	0.0003 ***
	Loneliness[a]	0.012	0.020	0.591	0.555
	Gender[b]	0.068	0.039	1.737	0.083
Negative individual events[v]					
Intercept		0.310	0.076	4.083	5.01e-05 ***
	Loneliness[a]	-0.022	0.024	-0.916	0.360
	Gender[b]	0.012	0.047	0.264	0.792
Positive interpersonal events[w]					
Intercept		0.001	0.052	0.014	0.989
	Loneliness[a]	-0.001	0.017	-0.013	0.990
	Gender[b]	0.098	0.032	3.064	0.002**
Negative interpersonal events[x]					
Intercept		0.236	0.080	2.935	0.003**
	Loneliness[a]	0.061	0.025	2.415	0.016 *
	Gender[b]	0.053	0.049	1.084	0.279
Positive collective event[y]					
Intercept		0.129	0.048	2.682	0.008**
	Loneliness[a]	0.008	0.015	0.549	0.583
	Gender[b]	-0.004	0.030	-0.142	0.887
Negative collective events[z]					
Intercept		0.129	0.068	1.906	0.057
	Loneliness[a]	0.005	0.021	0.253	0.800
	Gender[b]	0.053	0.042	1.278	0.202

*p<0.05, **p<0.01, ***p<0.0001

[a] Loneliness z-scores

[b] 1=Male, 2=Female

[c] Overall model: $F(2,385)$= 1.099, p = 0.334, *adjusted* R^2=0.001

[d] Overall model: $F(2,385)$= 0.472, p = 0.624, *adjusted* R^2=-0.003

[e] Overall model: $F(2,385)$= 0.286, p = 0.751, *adjusted* R^2=-0.004
[f] Overall model: $F(2,385)$= 2.360, p = 0.096, *adjusted* R^2=0.007
[g] Overall model: $F(2,385)$= 0.142, p = 0.868, *adjusted* R^2=-0.004
[h] Overall model: $F(2,385)$= 1.135, p = 0.332, *adjusted* R^2=0.001
[I] Overall model: $F(2,397)$= 0.518, p = 0.596, *adjusted* R^2=-0.002
[j] Overall model: $F(2,397)$= 7.700, p = 0.0005, *adjusted* R^2=0.032
[k] Overall model: $F(2, 397)$= 1.695, p = 0.185, *adjusted* R^2=0.003
[l] Overall model: $F(2, 397)$= 0.270, p = 0.764, *adjusted* R^2=-0.004
[m] Overall model: $F(2, 397)$= 0.913, p = 0.402, *adjusted* R^2=-0.001
[n] Overall model: $F(2, 397)$= 0.482, p = 0.618, *adjusted* R^2=-0.003
[o] Overall model: $F(2, 1193)$= 1.779, p = 0.169, *adjusted* R^2=0.001
[p] Overall model: $F(2, 1193)$= 0.421, p = 0.657, *adjusted* R^2=-0.001
[q] Overall model: $F(2, 1193)$= 2.484, p = 0.084, *adjusted* R^2=0.002
[r] Overall model: $F(2, 1193)$= 0.108, p = 0.897, *adjusted* R^2=-0.001
[s] Overall model: $F(2, 1193)$= 2.515, p = 0.081, *adjusted* R^2=0.003
[t] Overall model: $F(2, 1193)$= 0.931, p = 0.394, *adjusted* R^2=-0.001
[u] Overall model: $F(2, 645)$= 1.661, p = 0.191, *adjusted* R^2=0.002
[v] Overall model: $F(2, 645)$= 0.460, p = 0.632, *adjusted* R^2=-0.002
[w] Overall model: $F(2, 645)$= 4.696, p = 0.009, *adjusted* R^2=0.011
[x] Overall model: $F(2, 645)$= 3.450, p = 0.032, *adjusted* R^2=0.008
[y] Overall model: $F(2, 645)$= 0.163, p = 0.850, *adjusted* R^2=-0.003
[z] Overall model: $F(2, 645)$= 0.842, p = 0.431, *adjusted* R^2=-0.001

Table S7: Descriptive statistics for DANVA-2 scores in different age groups and genders for the two framing conditions

Age Group	Gender	Framing Condition		N	Min.	Max.	Mean	SD
16-24	Male	Non-social	Loneliness[a]	277	-2.09	2.11	0.27	0.97
			Full set of faces for all emotions	277	9.00	24.00	17.96	2.76
			Low intensity faces for all emotions	277	2.00	12.00	7.48	1.74
			Fear (low intensity only)	260	1.00	3.00	1.85	.64
			Anger (low intensity only)	221	1.00	3.00	1.59	.72
			Sadness (low intensity only)	275	1.00	3.00	2.36	.65
			Happiness (low intensity only)	275	1.00	3.00	2.14	.72
		Social	Loneliness[a]	278	-1.87	2.11	0.16	0.94
			Full set of faces for all emotions	278	1.00	23.00	17.30	3.23
			Low intensity faces for all emotions	277	2.00	11.00	7.11	1.80
			Fear (low intensity only)	247	1.00	3.00	1.79	.69
			Anger (low intensity only)	222	1.00	3.00	1.53	.68
			Sadness (low intensity only)	270	1.00	3.00	2.29	.677
			Happiness (low intensity only)	272	1.00	3.00	2.09	.76
	Female	Non-social	Loneliness[a]	504	-1.43	2.11	0.19	0.94
			Full set of faces for all emotions	504	8.00	23.00	18.60	2.77
			Low intensity faces for all emotions	504	3.00	12.00	7.79	1.79
			Fear (low intensity only)	472	1.00	3.00	1.84	.64
			Anger (low intensity only)	411	1.00	3.00	1.66	.73
			Sadness (low intensity only)	499	1.00	3.00	2.51	.64
			Happiness (low intensity only)	499	1.00	3.00	2.26	.71
		Social	Loneliness[a]	511	-1.87	2.11	0.22	0.94
			Full set of faces for all emotions	511	6.00	23.00	18.63	2.69
			Low intensity faces for all emotions	511	2.00	12.00	7.83	1.64
			Fear (low intensity only)	472	1.00	3.00	1.83	.67
			Anger (low intensity only)	440	1.00	3.00	1.63	.73
			Sadness (low intensity only)	506	1.00	3.00	2.55	.62
			Happiness (low intensity only)	509	1.00	3.00	2.23	.72
25-34	Male	Non-social	Loneliness[a]	488	-1.43	2.11	0.21	0.96
			Full set of faces for all emotions	488	7.00	23.00	18.00	2.96
			Low intensity faces for all emotions	488	1.00	12.00	7.52	1.76
			Fear (low intensity only)	442	1.00	3.00	1.79	.71
			Anger (low intensity only)	375	1.00	3.00	1.62	.71
			Sadness (low intensity only)	475	1.00	3.00	2.48	.67
			Happiness (low intensity only)	483	1.00	3.00	2.26	.68

		Social	Loneliness[a]	466	-2.09	2.11	0.22	1.01
			Full set of faces for all emotions	466	7.00	23.00	17.93	2.60
			Low intensity faces for all emotions	466	3.00	11.00	7.39	1.66
			Fear (low intensity only)	408	1.00	3.00	1.79	.679
			Anger (low intensity only)	356	1.00	3.00	1.589	.68
			Sadness (low intensity only)	461	1.00	3.00	2.44	.67
			Happiness (low intensity only)	459	1.00	3.00	2.25	.69
	Female	Non-social	Loneliness[a]	969	-2.09	2.11	-0.01	0.95
			Full set of faces for all emotions	969	5.00	24.00	18.94	2.60
			Low intensity faces for all emotions	969	2.00	12.00	8.01	1.72
			Fear (low intensity only)	907	1.00	3.00	1.84	.69
			Anger (low intensity only)	820	1.00	3.00	1.71	.76
			Sadness (low intensity only)	961	1.00	3.00	2.58	.60
			Happiness (low intensity only)	957	1.00	3.00	2.30	.67
		Social	Loneliness[a]	936	-1.65	2.11	-0.03	0.92
			Full set of faces for all emotions	936	5.00	24.00	19.10	2.57
			Low intensity faces for all emotions	936	2.00	12.00	8.16	1.67
			Fear (low intensity only)	879	1.00	3.00	1.88	.69
			Anger (low intensity only)	797	1.00	3.00	1.75	.75
			Sadness (low intensity only)	930	1.00	3.00	2.64	.57
			Happiness (low intensity only)	926	1.00	3.00	2.31	.68
35-64	Male	Non-social	Loneliness[a]	2151	-2.09	2.11	0.15	1.03
			Full set of faces for all emotions	2151	2.00	24.00	17.66	3.02
			Low intensity faces for all emotions	2151	1.00	12.00	7.68	1.77
			Fear (low intensity only)	1951	1.00	3.00	1.86	.69
			Anger (low intensity only)	1674	1.00	3.00	1.70	.74
			Sadness (low intensity only)	2097	1.00	3.00	2.41	.69
			Happiness (low intensity only)	2127	1.00	3.00	2.35	.67
		Social	Loneliness[a]	2217	-1.87	2.11	0.10	4.57
			Full set of faces for all emotions	2217	3.00	24.00	17.897	1.01
			Low intensity faces for all emotions	2217	1.00	12.00	7.81	1.76
			Fear (low intensity only)	2029	1.00	3.00	1.89	.70
			Anger (low intensity only)	1744	1.00	3.00	1.74	.75
			Sadness (low intensity only)	2178	1.00	3.00	2.42	.68
			Happiness (low intensity only)	2202	1.00	3.00	2.34	.68
	Female	Non-social	Loneliness[a]	4754	-2.09	2.11	-0.04	1.00
			Full set of faces for all emotions	4754	3.00	24.00	18.42	2.74
			Low intensity faces for all emotions	4754	1.00	12.00	8.04	1.69
			Fear (low intensity only)	4468	1.00	3.00	1.94	.70
			Anger (low intensity only)	3836	1.00	3.00	1.76	.76
			Sadness (low intensity only)	4676	1.00	3.00	2.55	.64

			Happiness (low intensity only)	4698	1.00	3.00	2.32	.67
		Social	Loneliness[a]	4571	-2.09	2.11	0.01	0.99
			Full set of faces for all emotions	4571	4.00	24.00	18.61	2.652
			Low intensity faces for all emotions	4571	1.00	12.00	8.17	1.67
			Fear (low intensity only)	4300	1.00	3.00	1.98	.69
			Anger (low intensity only)	3732	1.00	3.00	1.77	.75
			Sadness (low intensity only)	4522	1.00	3.00	2.57	.62
			Happiness (low intensity only)	4526	1.00	3.00	2.33	.67
65 or over	Male	Non-social	Loneliness[a]	623	-1.65	2.11	-0.11	1.04
			Full set of faces for all emotions	623	4.00	24.00	16.82	3.07
			Low intensity faces for all emotions	623	2.00	12.00	7.48	1.85
			Fear (low intensity only)	563	1.00	3.00	1.86	.68
			Anger (low intensity only)	490	1.00	3.00	1.71	.76
			Sadness (low intensity only)	598	1.00	3.00	2.23	.71
			Happiness (low intensity only)	614	1.00	3.00	2.35	.71
		Social	Loneliness[a]	659	-1.87	2.11	-0.21	0.97
			Full set of faces for all emotions	659	7.00	23.00	17.07	2.86
			Low intensity faces for all emotions	659	2.00	12.00	7.65	1.77
			Fear (low intensity only)	617	1.00	3.00	1.86	.66
			Anger (low intensity only)	529	1.00	3.00	1.78	.77
			Sadness (low intensity only)	641	1.00	3.00	2.25	.73
			Happiness (low intensity only)	655	1.00	3.00	2.31	.70
	Female	Non-social	Loneliness[a]	1323	-2.09	2.11	-0.21	0.95
			Full set of faces for all emotions	1323	5.00	24.00	17.72	2.72
			Low intensity faces for all emotions	1323	2.00	12.00	7.86	1.68
			Fear (low intensity only)	1276	1.00	3.00	1.98	.66
			Anger (low intensity only)	1047	1.00	3.00	1.77	.76
			Sadness (low intensity only)	1302	1.00	3.00	2.40	.69
			Happiness (low intensity only)	1298	1.00	3.00	2.23	.71
		Social	Loneliness[a]	1327	-2.09	2.11	-0.27	0.97
			Full set of faces for all emotions	1327	2.00	24.00	17.76	2.71
			Low intensity faces for all emotions	1327	1.00	12.00	7.91	1.70
			Fear (low intensity only)	1276	1.00	3.00	1.96	.65
			Anger (low intensity only)	1074	1.00	3.00	1.77	.75
			Sadness (low intensity only)	1298	1.00	3.00	2.40	.68
			Happiness (low intensity only)	1303	1.00	3.00	2.28	.69

N=sample size, Min.=Minimum, Max.=Maximum, SD=Standard Deviation
[a] z-scores of total scores on the four-item UCLA scale.

Table S8: Linear regression model predicting total and low-intensity-only Emotion Recognition (DANVA-2) scores for all emotions combined in the different age groups.

Dependent variable			Estimate	SE	t-value	p-value
16-24 year-olds						
Total DANVA-2 scores[d]						
	Intercept		17.125	0.339	50.565	< 2e-16 ***
		Loneliness[a]	-0.600	0.238	-2.513	0.012*
		Framing condition[b]	-0.275	0.146	-1.880	0.060
		Gender[c]	0.973	0.149	6.516	9.73e-11 ***
		Loneliness * Condition	0.247	0.151	1.639	0.102
Low intensity DANVA-2 scores[e]						
	Intercept		6.991	0.208	33.641	< 2e-16 ***
		Loneliness[a]	-0.231	0.146	-1.586	0.113
		Framing condition[b]	-0.120	0.090	-1.342	0.180
		Gender[c]	0.513	0.092	5.600	2.52e-08 ***
		Loneliness * Condition	0.073	0.093	0.793	0.428
25-34 year olds						
Total DANVA-2 scores[f]						
	Intercept		16.820	0.236	71.346	<2e-16 ***
		Loneliness[a]	0.281	0.163	1.733	0.083
		Framing condition[b]	0.096	0.099	0.964	0.335
		Gender[c]	1.026	0.106	9.688	<2e-16 ***
		Loneliness * Condition	-0.257	0.103	-2.487	0.013 *
Low intensity DANVA-2 scores[g]						
	Intercept		6.740	0.151	44.651	< 2e-16 ***
		Loneliness[a]	0.246	0.104	2.371	0.018 *
		Framing condition[b]	0.070	0.064	1.093	0.273
		Gender[c]	0.621	0.068	9.150	< 2e-16 ***

	Loneliness * Condition	-0.181	0.066	-2.732	0.006 **
35-64 year olds					
Total DANVA-2 score[h]					
Intercept		16.755	0.115	145.967	< 2e-16 ***
	Loneliness[a]	-0.058	0.074	-0.785	0.433
	Framing condition[b]	0.207	0.047	4.367	1.27e-05 ***
	Gender[c]	0.725	0.051	14.205	< 2e-16 ***
	Loneliness * Condition	-0.036	0.047	-0.765	0.444
Low intensity DANVA-2 scores[i]					
Intercept		7.203	0.071	101.982	<2e-16 ***
	Loneliness[a]	-0.047	0.045	-1.029	0.303
	Framing condition[b]	0.126	0.029	4.302	1.71e-05 ***
	Gender[c]	0.356	0.031	11.346	<2e-16 ***
	Loneliness * Condition	-0.003	0.029	-0.114	0.909
65+ years old					
Total DANVA-2 scores[i]					
Intercept		16.001	0.216	74.117	< 2e-16 ***
	Loneliness[a]	-0.104	0.144	-0.721	0.471
	Framing condition[b]	0.096	0.091	1.053	0.292
	Gender[c]	0.783	0.095	8.223	2.67e-16 ***
	Loneliness * Condition	-0.017	0.091	-0.183	0.855
Low intensity DANVA-2 scores[k]					
Intercept		7.115	0.134	53.239	< 2e-16 ***
	Loneliness[a]	-0.078	0.089	-0.875	0.382
	Framing condition[b]	0.089	0.057	1.566	0.117
	Gender[c]	0.313	0.059	5.311	1.15e-07 ***
	Loneliness * Condition	0.017	0.056	0.307	0.759

*p<0.05, **p<0.01, ***p<0.0001

[a] Loneliness measured using 4-item UCLA loneliness measure transformed into z-scores

[b] 1=non-social framing, 2=social framing

[c] 1=Male, 2=Female

[d] Overall model: $F(4,1565)$=14.40, p = 1.505e-11***, *adjusted* R^2=0.033

[e] Overall model: $F(4,1564)$=10.19, p = 3.829e-08***, *adjusted* R^2=0.023

[f] Overall model: $F(4,2854)$= 27.68, p = < 2.2e-16***, *adjusted* R^2=0.034

[g] Overall model: $F(4,2854)$= 23.89, p = < 2.2e-16***, *adjusted* R^2=0.031

[h] Overall model: $F(4,13688)$=62.76, p = <2.2e-16 ***, *adjusted* R^2=0.018

[i] Overall model: $F(4,13688)$=41.05, p = < 2.2e-16***, *adjusted* R^2=0.012

[j] Overall model: $F(4,3927)$=19.70, p = 4.572e-16***, *adjusted* R^2=0.019

[k] Overall model: $F(4,3927)$=8.72, p = 5.248e-07***, *adjusted* R^2=0.008

Table S9: Linear regression model predicting emotion recognition (DANVA-2) scores for faces showing different low intensity emotions for all the age groups.

Covariates		Estimate	SE	t-value	p-value
		16-24 year olds			
		Low intensity DANVA-2 scores – Fear[d]			
Intercept		1.845	0.082	22.540	<2e-16 ***
	Loneliness[a]	0.026	0.058	0.459	0.646
	Framing condition[b]	-0.022	0.035	-0.626	0.531
	Gender[c]	0.009	0.036	0.245	0.806
	Loneliness * Condition	-0.007	0.037	-0.187	0.851
		Low intensity DANVA-2 scores – Anger[e]			
Intercept		1.581	0.096	16.551	<2e-16 ***
	Loneliness[a]	-0.118	0.067	-1.763	0.078
	Framing condition[b]	-0.060	0.041	-1.465	0.143
	Gender[c]	0.079	0.042	1.875	0.061
	Loneliness * Condition	0.062	0.042	1.471	0.142
		Low intensity DANVA-2 scores – Sad[f]			
Intercept		2.134	0.077	27.644	< 2e-16 ***
	Loneliness[a]	-0.045	0.054	-0.835	0.404
	Framing condition[b]	-0.004	0.033	-0.106	0.916
	Gender[c]	0.200	0.034	5.847	6.1e-09 ***
	Loneliness * Condition	0.022	0.034	0.647	0.518
		Low intensity DANVA-2 scores – Happy[g]			
Intercept		2.058	0.087	23.777	< 2e-16 ***
	Loneliness[a]	-0.068	0.061	-1.108	0.268
	Framing condition[b]	-0.037	0.037	-0.989	0.323
	Gender[c]	0.127	0.038	3.329	0.0008***
	Loneliness * Condition	0.004	0.039	0.114	0.910
		25-34 year-olds			
		Low intensity DANVA-2 scores – Fear[h]			
Intercept		1.675	0.064	26.102	< 2e-16 ***
	Loneliness[a]	0.116	0.044	2.631	0.009**

	Framing condition[b]	0.028	0.027	1.047	0.295
	Gender[c]	0.071	0.029	2.449	0.014*
	Loneliness * Condition	-0.072	0.028	-2.555	0.011*
	Low intensity DANVA-2 scores – Anger[i]				
Intercept		1.459	0.073	19.855	< 2e-16 ***
	Loneliness	0.005	0.050	0.093	0.926
	Framing condition[a]	0.010	0.030	0.344	0.731
	Gender[b]	0.128	0.033	3.864	0.0001***
	Loneliness * Condition	-0.007	0.032	-0.206	0.837
	Low intensity DANVA-2 scores – Sad[j]				
Intercept		2.270	0.055	41.077	< 2e-16 ***
	Loneliness	0.067	0.038	1.753	0.080
	Framing condition[a]	0.028	0.023	1.196	0.232
	Gender[b]	0.151	0.025	6.093	1.26e-09 ***
	Loneliness * Condition	-0.052	0.024	-2.151	0.032 *
	Low intensity DANVA-2 scores – Happy[k]				
Intercept		2.207	0.061	36.333	<2e-16 ***
	Loneliness	-0.013	0.042	-0.305	0.761
	Framing condition[a]	0.001	0.026	0.021	0.983
	Gender[b]	0.050	0.027	1.815	0.070
	Loneliness * Condition	-0.005	0.027	-0.179	0.858
	35-64 year olds				
	Low intensity DANVA-2 scores – Fear[l]				
Intercept		1.736	0.030	57.947	< 2e-16 ***
	Loneliness	0.013	0.019	0.692	0.489
	Framing condition[a]	0.039	0.012	3.205	0.001 **
	Gender[b]	0.084	0.013	6.280	3.5e-10 ***
	Loneliness * Condition	-0.009	0.012	-0.697	0.486
	Low intensity DANVA-2 scores – Anger[m]				
Intercept		1.645	0.035	47.157	< 2e-16 ***
	Loneliness	-0.044	0.022	-1.973	0.049 *
	Framing condition[a]	0.020	0.014	1.369	0.171
	Gender[b]	0.043	0.016	2.788	0.005 **

	Loneliness * Condition	0.021	0.014	1.483	0.138
Low intensity DANVA-2 scores – Sad[n]					
Intercept		2.236	0.027	82.606	<2e-16 ***
	Loneliness	-0.011	0.017	-0.632	0.527
	Framing condition[a]	0.024	0.011	2.127	0.033 *
	Gender[b]	0.145	0.012	12.017	<2e-16 ***
	Loneliness * Condition	-0.003	0.011	-0.263	0.793
Low intensity DANVA-2 scores – Happy[o]					
Intercept		2.359	0.028	84.873	<2e-16 ***
	Loneliness	-0.025	0.018	-1.393	0.164
	Framing condition[a]	0.005	0.011	0.458	0.647
	Gender[b]	-0.019	0.012	-1.542	0.123
	Loneliness * Condition	0.002	0.011	0.219	0.826
65 years and over					
Low intensity DANVA-2 scores – Fear[p]					
Intercept		1.775	0.053	33.583	< 2e-16 ***
	Loneliness	-0.002	0.035	-0.052	0.958
	Framing condition[a]	-0.014	0.022	-0.654	0.513
	Gender[b]	0.107	0.023	4.592	4.53e-06 ***
	Loneliness * Condition	-0.001	0.022	-0.017	0.986
Low intensity DANVA-2 scores – Anger[q]					
Intercept		1.679	0.066	25.549	<2e-16 ***
	Loneliness	-0.041	0.044	-0.923	0.356
	Framing condition[a]	0.031	0.028	1.132	0.258
	Gender[b]	0.022	0.029	0.773	0.439
	Loneliness * Condition	0.024	0.028	0.889	0.374
Low intensity DANVA-2 scores – Sad[r]					
Intercept		2.075	0.055	37.897	< 2e-16 ***
	Loneliness	0.021	0.036	0.577	0.564
	Framing condition[a]	0.001	0.023	0.021	0.983
	Gender[b]	0.161	0.024	6.673	2.87e-11 ***
	Loneliness * Condition	-0.027	0.023	-1.158	0.247

Low intensity DANVA-2 scores – Happy[s]					
Intercept		2.365	0.055	43.289	< 2e-16 ***
	Loneliness	-0.017	0.036	-0.475	0.635
	Framing condition[a]	0.018	0.023	0.759	0.448
	Gender[b]	-0.069	0.024	-2.885	0.004**
	Loneliness * Condition	-0.005	0.0230	-0.217	0.828

*p<0.05, **p<0.01, ***p<0.0001

[a] Loneliness measured using 4-item UCLA loneliness measure transformed into z-scores

[b] 1=non-social framing, 2=social framing

[c] 1=Male, 2=Female

[d] Overall model: $F(4,1446)$=0.334, p = 0.855, *adjusted* R^2=-0.002

[e] Overall model: $F(4,1289)$=2.164, p = 0.071, *adjusted* R^2=0.003

[f] Overall model: $F(4,1545)$=8.866, p = 4.463e-07, *adjusted* R^2=0.020

[g] Overall model: $F(4,1550)$=5.528, p = 0.002, *adjusted* R^2=0.012

[h] Overall model: $F(4,2631)$=3.416, p = 0.009**, *adjusted* R^2=0.004

[i] Overall model: $F(4, 2343)$= 3.896, p = 0.004**, *adjusted* R^2=0.005

[j] Overall model: $F(4, 2822)$= 11.44, p = 3.275e-09***, *adjusted* R^2=0.015

[k] Overall model: $F(4, 2820)$= 1.557, p = 0.183, *adjusted* R^2=0.001

[l] Overall model: $F(4, 12743)$= 12.33, p = 5.196e-10, *adjusted* R^2=0.004

[m] Overall model: $F(4, 10981)$= 3.973, p = 0.003, *adjusted* R^2=0.001

[n] Overall model: $F(4, 13468)$= 40.24, p = < 2.2e-16, *adjusted* R^2=0.012

[o] Overall model: $F(4, 13548)$= 3.943, p = 0.003, *adjusted* R^2=0.001

[p] Overall model: $F(4, 3727)$= 5.463, p = 0.0002, *adjusted* R^2=0.005

[q] Overall model: $F(4, 3135)$= 0.599, p = 0.664, *adjusted* R^2=-0.001

[r] Overall model: $F(4, 3834)$= 12.39, p = 5.166e-10, *adjusted* R^2=0.012

[s] Overall model: $F(4, 3865)$= 3.367, p = 0.009, *adjusted* R^2=0.002

R Code for analysis

Social memory task

```
# set working directory, load data and create dataframe for analysis------
getwd() #get path for directory and then set it
setwd("")
data_socialmem = read.csv("social_memory_data.csv", header = T, sep = ',',
na.strings="NA")
data_socialmem[data_socialmem == -99] <- NA #change all -99 values to NA
data_SM <- as.data.frame(data_socialmem$ID)
colnames(data_SM)[colnames(data_SM)=="data_socialmem$ID"] <- "ID"
#demographics
data_SM$Gender <- data_socialmem$Gender # 1=Male, 2=Female, 3=Other,
4=Prefer not to say
data_SM$Age_continuous <- data_socialmem$Age_Open
data_SM$Age_originalCategories <- factor(data_socialmem$Age_Categories)
#Loneliness measure
data_SM$total_UCLA_freq <- data_socialmem$UCLA_Frequency_Total
data_SM$total_UCLA_freq <-
ifelse(data_SM$total_UCLA_freq!=0,data_SM$total_UCLA_freq,NA)#remove zero
scores
sd_freq <- sd(data_SM$total_UCLA_freq,na.rm=T)
mean_freq <- mean(data_SM$total_UCLA_freq,na.rm=T)
data_SM$Loneliness_freq_zscore <- cbind((data_SM$total_UCLA_freq -
mean_freq)/sd_freq)
#memory variables
data_SM$individual_positive <- data_socialmem$individual_positive
data_SM$individual_negative <- data_socialmem$individual_negative
data_SM$interpersonal_positive <- data_socialmem$interpersonal_positive
data_SM$interpersonal_negative <- data_socialmem$interpersonal_negative
data_SM$collective_positive <- data_socialmem$collective_positive
data_SM$collective_negative <- data_socialmem$collective_negative

#Missingness
data_SM <- subset(data_SM,data_SM$Age_originalCategories!=1) #remove <16
year olds
#remove participants with missing data for Age (continuous)
data_SM_subset <- data_SM[which(!is.na(data_SM$Age_continuous)),]
#remove ps missing gender data
data_SM_subset$Gender[which(data_SM_subset$Gender==3)] <- NA
data_SM_subset$Gender[which(data_SM_subset$Gender==4)] <- NA
data_SM_subset1 <- data_SM_subset[which(!is.na(data_SM_subset$Gender)),]
#remove participants missing loneliness/memory scores
data_SM_subset2 <-
data_SM_subset1[which(!is.na(data_SM_subset1$total_UCLA_freq)),]
data_SM_subset3 <-
data_SM_subset2[which(!is.na(data_SM_subset2$individual_positive)),]
data_SM_subset4 <-
data_SM_subset3[which(!is.na(data_SM_subset3$individual_negative)),]
```

```
data_SM_subset5 <-
data_SM_subset4[which(!is.na(data_SM_subset4$interpersonal_positive)),]
data_SM_subset6 <-
data_SM_subset5[which(!is.na(data_SM_subset5$interpersonal_negative)),]
data_SM_subset7 <-
data_SM_subset6[which(!is.na(data_SM_subset6$collective_positive)),]
data_SM_subset8 <-
data_SM_subset7[which(!is.na(data_SM_subset7$collective_negative)),]


data_SM_full<- data_SM_subset8

rm(data_SM_subset)
rm(data_SM_subset1)
rm(data_SM_subset2)
rm(data_SM_subset3)
rm(data_SM_subset4)
rm(data_SM_subset5)
rm(data_SM_subset6)
rm(data_SM_subset7)
rm(data_SM_subset8)

#collapse age categories
data_SM_full$Age_groups <- c() #create age groups 0=16-24, 1=25-34,2=35-64,
3=65+
#in master data age categories are 1=<16, 2=16-24, 3=25-34, 4=35-44, 5=45-54,
6=55-64, 7=65-74, 8=75+
for (i in 1:nrow(data_SM_full)) {
  if (data_SM_full$Age_originalCategories[i]==2) {
    data_SM_full$Age_groups[i] <- 0
  } else if (data_SM_full$Age_originalCategories[i]==3) {
    data_SM_full$Age_groups[i]<-1
  } else if
(data_SM_full$Age_originalCategories[i]==4|data_SM_full$Age_originalCategories[
i]==5|data_SM_full$Age_originalCategories[i]==6) {
    data_SM_full$Age_groups[i]<-2
  } else if
(data_SM_full$Age_originalCategories[i]==7|data_SM_full$Age_originalCategories[
i]==8) {
    data_SM_full$Age_groups[i]<- 3
  } else if
(data_SM_full$Age_originalCategories[i]==NA|data_SM_full$Age_originalCategori
es[i]==1) {
    data_SM_full$Age_groups[i]<- NA
  }
}
rm (i)
data_SM_full$Age_groups <- factor(data_SM_full$Age_groups,ordered=F)
```

```
#Demographics
table(data_SM_full$Gender)
summary(data_SM_full$Age_continuous)
sd(data_SM_full$Age_continuous)

#ANALYSIS memory task data
#full sample
#Overall model
#First need to rearrange data into long form see http://www.cookbook-r.com/Manipulating_data/Converting_data_between_wide_and_long_format/
library(tidyr)
library(lmerTest)
library(lme4)
data_SM_full$ID <- factor(data_SM_full$ID)
data_SM_full_long <- gather(data_SM_full, event_type, recall_score, individual_positive:collective_negative, factor_key=TRUE)
head(data_SM_full_long)
#then code two new variables denoting event type (individual, interpersonal, collective) and event valence (positive or negative)
#first, type of event
data_SM_full_long = cbind(data_SM_full_long,NA)
colnames(data_SM_full_long)[ncol(data_SM_full_long)]="event"
data_SM_full_long$event[which(data_SM_full_long$event_type=="individual_positive")]=1 # individual=1, interpersonal=2, collective=3
data_SM_full_long$event[which(data_SM_full_long$event_type=="individual_negative")]=1
data_SM_full_long$event[which(data_SM_full_long$event_type=="interpersonal_positive")]=2
data_SM_full_long$event[which(data_SM_full_long$event_type=="interpersonal_negative")]=2
data_SM_full_long$event[which(data_SM_full_long$event_type=="collective_positive")]=3
data_SM_full_long$event[which(data_SM_full_long$event_type=="collective_negative")]=3
#second, event valence
data_SM_full_long = cbind(data_SM_full_long,NA)
colnames(data_SM_full_long)[ncol(data_SM_full_long)]="valence"
data_SM_full_long$valence[which(data_SM_full_long$event_type=="individual_positive")]=2 # negative=1, positive=2
data_SM_full_long$valence[which(data_SM_full_long$event_type=="individual_negative")]=1
data_SM_full_long$valence[which(data_SM_full_long$event_type=="interpersonal_positive")]=2
data_SM_full_long$valence[which(data_SM_full_long$event_type=="interpersonal_negative")]=1
data_SM_full_long$valence[which(data_SM_full_long$event_type=="collective_positive")]=2
data_SM_full_long$valence[which(data_SM_full_long$event_type=="collective_negative")]=1
```

```
#hierarchical linear model predicting recall scores from lonelines z scores * Age
(continuous) *event * valence + Gender,
#nesting these variables within participant ID
recallModel <-
lmer(recall_score~Loneliness_freq_zscore*event*valence*Age_continuous+Gender+
(1|ID),data=data_SM_full_long)
summary(recallModel)
#look at valences separately
recallModel_negative <-
lmer(recall_score~Loneliness_freq_zscore*event*valence*Age_continuous+Gender+
(1|ID),data=data_SM_full_long, subset=valence==1)
summary(recallModel_negative)
recallModel_positive <-
lmer(recall_score~Loneliness_freq_zscore*event*valence*Age_continuous+Gender+
(1|ID),data=data_SM_full_long, subset=valence==2)
summary(recallModel_positive)
#look at event type separately
recallModel_indiv <-
lmer(recall_score~Loneliness_freq_zscore*event*valence*Age_continuous+Gender+
(1|ID),data=data_SM_full_long, subset=event==1)
summary(recallModel_indiv)
recallModel_inter <-
lmer(recall_score~Loneliness_freq_zscore*event*valence*Age_continuous+Gender+
(1|ID),data=data_SM_full_long, subset=event==2)
summary(recallModel_inter)
recallModel_collect <-
lmer(recall_score~Loneliness_freq_zscore*event*valence*Age_continuous+Gender+
(1|ID),data=data_SM_full_long, subset=event==3)
summary(recallModel_collect)

#Next, look at different events types/valences separately: number of different types of
events correctly recalled are regressed on loneliness*Age+Gender (age as a
continuous variable)
ind_pos <-
lm(data_SM_full$individual_positive~data_SM_full$Loneliness_freq_zscore*data_S
M_full$Age_continuous+data_SM_full$Gender)
summary(ind_pos)
ind_neg <-
lm(data_SM_full$individual_negative~data_SM_full$Loneliness_freq_zscore*data_
SM_full$Age_continuous+data_SM_full$Gender)
summary(ind_neg)
inter_pos <-
lm(data_SM_full$interpersonal_positive~data_SM_full$Loneliness_freq_zscore*data
_SM_full$Age_continuous+data_SM_full$Gender)
summary(inter_pos)
inter_neg <-
lm(data_SM_full$interpersonal_negative~data_SM_full$Loneliness_freq_zscore*dat
a_SM_full$Age_continuous+data_SM_full$Gender)
summary(inter_neg)
```

```
collect_pos <-
lm(data_SM_full$collective_positive~data_SM_full$Loneliness_freq_zscore*data_S
M_full$Age_continuous+data_SM_full$Gender)
summary(collect_pos)
collect_neg <-
lm(data_SM_full$collective_negative~data_SM_full$Loneliness_freq_zscore*data_S
M_full$Age_continuous+data_SM_full$Gender)
summary(collect_neg)


#each age group separately 0=16-24, 1=25-34,2=35-64, 3=65+
#16-24
ind_posYP <-
lm(data_SM_full$individual_positive~data_SM_full$Loneliness_freq_zscore+data_S
M_full$Gender,
            subset=data_SM_full$Age_groups==0)
summary(ind_posYP)
ind_negYP <-
lm(data_SM_full$individual_negative~data_SM_full$Loneliness_freq_zscore+data_
SM_full$Gender,
            subset=data_SM_full$Age_groups==0)
summary(ind_negYP)
inter_posYP <-
lm(data_SM_full$interpersonal_positive~data_SM_full$Loneliness_freq_zscore+data
_SM_full$Gender,
              subset=data_SM_full$Age_groups==0)
summary(inter_posYP)
inter_negYP <-
lm(data_SM_full$interpersonal_negative~data_SM_full$Loneliness_freq_zscore+dat
a_SM_full$Gender,
              subset=data_SM_full$Age_groups==0)
summary(inter_negYP)
collect_posYP <-
lm(data_SM_full$collective_positive~data_SM_full$Loneliness_freq_zscore+data_S
M_full$Gender,
                subset=data_SM_full$Age_groups==0)
summary(collect_posYP)
collect_negYP <-
lm(data_SM_full$collective_negative~data_SM_full$Loneliness_freq_zscore+data_S
M_full$Gender,
                subset=data_SM_full$Age_groups==0)
summary(collect_negYP)
#25-34
ind_pos2534 <-
lm(data_SM_full$individual_positive~data_SM_full$Loneliness_freq_zscore+data_S
M_full$Gender,
           subset=data_SM_full$Age_groups==1)
summary(ind_pos2534)
```

```
ind_neg2534 <-
lm(data_SM_full$individual_negative~data_SM_full$Loneliness_freq_zscore+data_
SM_full$Gender,
        subset=data_SM_full$Age_groups==1)
summary(ind_neg2534)
inter_pos2534 <-
lm(data_SM_full$interpersonal_positive~data_SM_full$Loneliness_freq_zscore+data
_SM_full$Gender,
        subset=data_SM_full$Age_groups==1)
summary(inter_pos2534)
inter_neg2534 <-
lm(data_SM_full$interpersonal_negative~data_SM_full$Loneliness_freq_zscore+dat
a_SM_full$Gender,
        subset=data_SM_full$Age_groups==1)
summary(inter_neg2534)
collect_pos2534 <-
lm(data_SM_full$collective_positive~data_SM_full$Loneliness_freq_zscore+data_S
M_full$Gender,
         subset=data_SM_full$Age_groups==1)
summary(collect_pos2534)
collect_neg2534 <-
lm(data_SM_full$collective_negative~data_SM_full$Loneliness_freq_zscore+data_S
M_full$Gender,
         subset=data_SM_full$Age_groups==1)
summary(collect_neg2534)
#age categories 35-64
ind_pos3564 <-
lm(data_SM_full$individual_positive~data_SM_full$Loneliness_freq_zscore+data_S
M_full$Gender,
        subset=data_SM_full$Age_groups==2)
summary(ind_pos3564)
ind_neg3564 <-
lm(data_SM_full$individual_negative~data_SM_full$Loneliness_freq_zscore+data_
SM_full$Gender,
        subset=data_SM_full$Age_groups==2)
summary(ind_neg3564)
inter_pos3564 <-
lm(data_SM_full$interpersonal_positive~data_SM_full$Loneliness_freq_zscore+data
_SM_full$Gender,
         subset=data_SM_full$Age_groups==2)
summary(inter_pos3564)
inter_neg3564 <-
lm(data_SM_full$interpersonal_negative~data_SM_full$Loneliness_freq_zscore+dat
a_SM_full$Gender,
         subset=data_SM_full$Age_groups==2)
summary(inter_neg3564)
collect_pos3564 <-
lm(data_SM_full$collective_positive~data_SM_full$Loneliness_freq_zscore+data_S
M_full$Gender,
          subset=data_SM_full$Age_groups==2)
```

```
summary(collect_pos3564)
collect_neg3564 <-
lm(data_SM_full$collective_negative~data_SM_full$Loneliness_freq_zscore+data_S
M_full$Gender,
          subset=data_SM_full$Age_groups==2)
summary(collect_neg3564)
#age categories
#65+
ind_pos65 <-
lm(data_SM_full$individual_positive~data_SM_full$Loneliness_freq_zscore+data_S
M_full$Gender,
         subset=data_SM_full$Age_groups==3)
summary(ind_pos65)
ind_neg65 <-
lm(data_SM_full$individual_negative~data_SM_full$Loneliness_freq_zscore+data_
SM_full$Gender,
         subset=data_SM_full$Age_groups==3)
summary(ind_neg65)
inter_pos65 <-
lm(data_SM_full$interpersonal_positive~data_SM_full$Loneliness_freq_zscore+data
_SM_full$Gender,
         subset=data_SM_full$Age_groups==3)
summary(inter_pos65)
inter_neg65 <-
lm(data_SM_full$interpersonal_negative~data_SM_full$Loneliness_freq_zscore+dat
a_SM_full$Gender,
          subset=data_SM_full$Age_groups==3)
summary(inter_neg65)
collect_pos65 <-
lm(data_SM_full$collective_positive~data_SM_full$Loneliness_freq_zscore+data_S
M_full$Gender,
           subset=data_SM_full$Age_groups==3)
summary(collect_pos65)
collect_neg65 <-
lm(data_SM_full$collective_negative~data_SM_full$Loneliness_freq_zscore+data_S
M_full$Gender,
           subset=data_SM_full$Age_groups==3)
summary(collect_neg65)
```

Emotion recognition task

```
#set working directory---------
getwd() #get path for directory and then set it
setwd("")

#FIRST DANVA analyses-----
#Load data file
data_csv = read.csv("BBC_loneliness_master_May2019.csv", header = T, sep = ',',
na.strings="NA")
data_csv[data_csv == -99] <- NA #change all -99 values to NA
```

```
#PREPARING THE DATA FOR ANALYSIS
#Create dataframe and rename variables to be used in this analysis
data <- as.data.frame(data_csv$ID)
colnames(data)[colnames(data)=="data_csv$ID"] <- "ID"
data$Gender <- data_csv$Gender # 1=Male, 2=Female, 3=Other, 4=Prefer not to say:
data$Age_continuous <- data_csv$Age_Open
data$Age_originalCategories <- factor(data_csv$Age_Categories) #age categories are
1=<16, 2=16-24, 3=25-34, 4=35-44, 5=45-54, 6=55-64, 7=65-74, 8=75+

#Nationality
data$Nationality_Living <- data_csv$Nationality_Living
data$Nationality_Born <- data_csv$Nationality_Born
data$nationality_match <-
ifelse(data$Nationality_Living==data$Nationality_Born,1,0) #1=living and born
nationality match

#Add framing condition
data$Condition <- data_csv$SocialFramingCondition #1=DANVA nonsocial,
2=DANVA social

#Add loneliness frequency
data$UCLA_Comp_Often <- data_csv$UCLA_Comp_Often
data$UCLA_Leftout_Often <- data_csv$UCLA_Leftout_Often
data$UCLA_Isolated_Often <- data_csv$UCLA_Isolated_Often
data$UCLA_Tune_Often <- data_csv$UCLA_Tune_Often #already reverse scored in
data_csv
#Loneliness measures - calculate  total scores for 4 item UCLA measure (total of
UCLA_Comp_Often + UCLA_Leftout_Often + UCLA_Isolated_Often+ reversed
scored UCLA_Tune_Often) using the formula
data$total_UCLA_freq <-
rowSums(cbind(data$UCLA_Comp_Often,data$UCLA_Leftout_Often,data$UCLA_
Isolated_Often,data$UCLA_Tune_Often), na.rm = T)
data$total_UCLA_freq <-
ifelse(data$total_UCLA_freq!=0,data$total_UCLA_freq,NA)#make zero scores NAs

#DANVA
data$total_DANVA_full <- rowSums(cbind(data_csv$DANVA_NS_1_Scored,
        data_csv$DANVA_NS_2_Scored,    data_csv$DANVA_NS_3_Scored,
        data_csv$DANVA_NS_4_Scored,
                data_csv$DANVA_NS_5_Scored,
        data_csv$DANVA_NS_6_Scored,    data_csv$DANVA_NS_7_Scored,
        data_csv$DANVA_NS_8_Scored,

data_csv$DANVA_NS_9_Scored,data_csv$DANVA_NS_10_Scored,
        data_csv$DANVA_NS_11_Scored,  data_csv$DANVA_NS_12_Scored,
                data_csv$DANVA_NS_13_Scored,
        data_csv$DANVA_NS_14_Scored,  data_csv$DANVA_NS_15_Scored,
        data_csv$DANVA_NS_16_Scored,
```

```
                    data_csv$DANVA_NS_17_Scored,
data_csv$DANVA_NS_18_Scored,  data_csv$DANVA_NS_19_Scored,
        data_csv$DANVA_NS_20_Scored,
                    data_csv$DANVA_NS_21_Scored,
        data_csv$DANVA_NS_22_Scored,  data_csv$DANVA_NS_23_Scored,
                    data_csv$DANVA_NS_24_Scored,
        data_csv$DANVA_S_1_Scored, data_csv$DANVA_S_2_Scored,
        data_csv$DANVA_S_3_Scored,
                    data_csv$DANVA_S_4_Scored,
        data_csv$DANVA_S_5_Scored,
                    data_csv$DANVA_S_6_Scored,
        data_csv$DANVA_S_7_Scored,      data_csv$DANVA_S_8_Scored,
        data_csv$DANVA_S_9_Scored,

data_csv$DANVA_S_10_Scored,data_csv$DANVA_S_11_Scored,
        data_csv$DANVA_S_12_Scored,     data_csv$DANVA_S_13_Scored,
                    data_csv$DANVA_S_14_Scored,
        data_csv$DANVA_S_15_Scored,     data_csv$DANVA_S_16_Scored,
                    data_csv$DANVA_S_17_Scored,
        data_csv$DANVA_S_18_Scored,     data_csv$DANVA_S_19_Scored,
        data_csv$DANVA_S_20_Scored,
                    data_csv$DANVA_S_21_Scored,
        data_csv$DANVA_S_22_Scored,     data_csv$DANVA_S_23_Scored,
data_csv$DANVA_S_24_Scored), na.rm=T)
                    #total_DANVA_nonsocial_full + total_DANVA_social_full
data$total_DANVA_full <-
ifelse(data$total_DANVA_full!=0,data$total_DANVA_full,NA) #change scores of
zero to NAs


#low intensity faces scores
data$DANVA_fear_lowinstensity <-
rowSums(cbind(data_csv$DANVA_NS_2_Scored, data_csv$DANVA_S_2_Scored,
data_csv$DANVA_NS_8_Scored, data_csv$DANVA_S_8_Scored,
                         data_csv$DANVA_NS_16_Scored,
data_csv$DANVA_S_16_Scored), na.rm=T) #low intensity for fear items 2, 8, 16 for
nonsocial and social framing conditions
data$DANVA_fear_lowinstensity <-
ifelse(data$DANVA_fear_lowinstensity!=0,data$DANVA_fear_lowinstensity,NA)
#change scores of zero to NAs
data$DANVA_anger_lowinstensity <-
rowSums(cbind(data_csv$DANVA_NS_5_Scored, data_csv$DANVA_S_5_Scored,
data_csv$DANVA_NS_12_Scored, data_csv$DANVA_S_12_Scored,
                         data_csv$DANVA_NS_20_Scored,
data_csv$DANVA_S_20_Scored), na.rm=T) #low intensity for anger items 5, 12, 20
for nonsocial and social framing conditions
data$DANVA_anger_lowinstensity <-
ifelse(data$DANVA_anger_lowinstensity!=0,data$DANVA_anger_lowinstensity,NA
) #change scores of zero to NAs
```

```
data$DANVA_sad_lowinstensity <-
rowSums(cbind(data_csv$DANVA_NS_13_Scored,
data_csv$DANVA_S_13_Scored, data_csv$DANVA_NS_14_Scored,
data_csv$DANVA_S_14_Scored,
                                  data_csv$DANVA_NS_17_Scored,
data_csv$DANVA_S_17_Scored), na.rm=T) #low intensity for sad items 13, 14, 17
for nonsocial and social framing conditions
data$DANVA_sad_lowinstensity <-
ifelse(data$DANVA_sad_lowinstensity!=0,data$DANVA_sad_lowinstensity,NA)
#change scores of zero to NAs

data$DANVA_happy_lowinstensity <-
rowSums(cbind(data_csv$DANVA_NS_4_Scored, data_csv$DANVA_S_4_Scored,
data_csv$DANVA_NS_7_Scored, data_csv$DANVA_S_7_Scored,
                                    data_csv$DANVA_NS_10_Scored,
data_csv$DANVA_S_10_Scored), na.rm=T) #low intensity for happy items 4, 7, 10
for nonsocial and social framing conditions
data$DANVA_happy_lowinstensity <-
ifelse(data$DANVA_happy_lowinstensity!=0,data$DANVA_happy_lowinstensity,N
A) #change scores of zero to NAs

data$DANVA_lowintensity <- rowSums(cbind(data$DANVA_fear_lowinstensity,
data$DANVA_anger_lowinstensity, data$DANVA_sad_lowinstensity,
                                data$DANVA_happy_lowinstensity), na.rm=T)
                               #total_DANVA_nonsocial_lowintensity +
total_DANVA_social_lowintensity for all emotions
data$DANVA_lowintensity <-
ifelse(data$DANVA_lowintensity!=0,data$DANVA_lowintensity,NA) #change
scores of zero to NAs

#add indicator of presentation error
data$present_error <- data_csv$SocialTasks_Errors_on_presentation

#Remove participants aged under 16 years-----
data <- subset(data,data$Age_originalCategories!=1)
#remove participants with missing data for Age (continuous) or Gender or DANVA
or Loneliness
data_subset <- data[which(!is.na(data$Age_continuous)),]
data_subset1 <- data_subset[which(!is.na(data_subset$Gender)),]
#3 & 4 removed due to smaller sample size of <250 (as opposed to >15,500 for M/F)
data_subset1$Gender[which(data_subset1$Gender==3)] <- NA
data_subset1$Gender[which(data_subset1$Gender==4)] <- NA
data_subset2 <- data_subset1[which(!is.na(data_subset1$Gender)),]
#remove participants missing loneliness/DANVA scores
data_subset3 <- data_subset2[which(!is.na(data_subset2$total_UCLA_freq)),]
data_subset4 <- data_subset3[which(!is.na(data_subset3$total_DANVA_full)),]
#remove participants for whom there was a presentation error in the social skills
tasks------
data_subset5 <- data_subset4[which(is.na(data_subset4$present_error)),]
data_full<- data_subset5
```

```
#collapse age categories
data_full$Age_groups <- c() #create age categories 0=16-24, 1=25-34,2=35-64,
3=65+
#in master data age categories are 1=<16, 2=16-24, 3=25-34, 4=35-44, 5=45-54,
6=55-64, 7=65-74, 8=75+
for (i in 1:nrow(data_full)) {
  if (data_full$Age_originalCategories[i]==2) {
    data_full$Age_groups[i] <- 0
  } else if (data_full$Age_originalCategories[i]==3) {
    data_full$Age_groups[i]<-1
  } else if
(data_full$Age_originalCategories[i]==4|data_full$Age_originalCategories[i]==5|dat
a_full$Age_originalCategories[i]==6) {
    data_full$Age_groups[i]<-2
  } else if
(data_full$Age_originalCategories[i]==7|data_full$Age_originalCategories[i]==8) {
    data_full$Age_groups[i]<- 3
  } else if
(data_full$Age_originalCategories[i]==NA|data_full$Age_originalCategories[i]==1)
{
    data_full$Age_groups[i]<- NA
  }
}
rm (i)
data_full$Age_groups <- factor(data_full$Age_groups,ordered=F)

sd_freq <- sd(data_full$total_UCLA_freq,na.rm=T)
mean_freq <- mean(data_full$total_UCLA_freq,na.rm=T)
data_full$Loneliness_freq_zscore <- cbind((data_full$total_UCLA_freq -
mean_freq)/sd_freq)


rm(data)
rm(data_csv)
rm(data_subset)
rm(data_subset1)
rm(data_subset2)
rm(data_subset3)
rm(data_subset4)
rm(data_subset5)
rm(mean_freq)
rm(sd_freq)

#Demographics
table(data_full$Gender)
summary(data_full$Age_continuous)
sd(data_full$Age_continuous)

#reliability
```

```
library(psych)
Ldata <-
cbind(data_full$UCLA_Comp_Often,data_full$UCLA_Leftout_Often,data_full$UC
LA_Isolated_Often)
alpha(Ldata)

#ANALYSIS------

#gender comparisons # 1=Male, 2=Female
L_gender <- lm(Loneliness_freq_zscore~Gender,data=data_full)
summary(L_gender)
danva_gender <- lm(total_DANVA_full~Gender,data=data_full)
summary(danva_gender)

#NB for the framing condition 1=DANVA nonsocial, 2=DANVA social
#in all analyses exclude participants with unspecified Gender
#for sensitivity analyses exclude participants with DANVA scores under 8 (3 SDs
below mean)
#FIRST DANVA
# DANVA Total scores & freq loneliness with age as covariate----
DANVAlonelinessfreq_agecont <-
lm(data_full$total_DANVA_full~data_full$Loneliness_freq_zscore*data_full$Condi
tion*data_full$Age_continuous+data_full$Gender) #age as continuous variable
summary(DANVAlonelinessfreq_agecont)
#sensitivity analysis - excluding DANVA <8
DANVAlonelinessfreq_agecont <-
lm(data_full$total_DANVA_full~data_full$Loneliness_freq_zscore*data_full$Condi
tion*data_full$Age_continuous+data_full$Gender,
                              subset=data_full$total_DANVA_full>=8) #age as continuous
variable
summary(DANVAlonelinessfreq_agecont)
#low intensity DANVA faces - combined scores
# DANVA Total low intensity scores & freq loneliness with  age as covariate----
lowDANVAlonelinessfreq <-
lm(data_full$DANVA_lowintensity~data_full$Loneliness_freq_zscore*data_full$Co
ndition*data_full$Age_continuous+data_full$Gender)
summary(lowDANVAlonelinessfreq)

#Different low intensity emotions separately
DANVAlonelinessfreq_fear <-
lm(data_full$DANVA_fear_lowinstensity~data_full$Loneliness_freq_zscore*data_fu
ll$Condition*data_full$Age_continuous+data_full$Gender) #age as continuous
variable
summary(DANVAlonelinessfreq_fear)
DANVAlonelinessfreq_anger <-
lm(data_full$DANVA_anger_lowinstensity~data_full$Loneliness_freq_zscore*data_
full$Condition*data_full$Age_continuous+data_full$Gender) #age as continuous
variable
summary(DANVAlonelinessfreq_anger)
```

```
DANVAlonelinessfreq_sad <-
lm(data_full$DANVA_sad_lowinstensity~data_full$Loneliness_freq_zscore*data_fu
ll$Condition*data_full$Age_continuous+data_full$Gender) #age as continuous
variable
summary(DANVAlonelinessfreq_sad)
DANVAlonelinessfreq_happy <-
lm(data_full$DANVA_happy_lowinstensity~data_full$Loneliness_freq_zscore*data
_full$Condition*data_full$Age_continuous+data_full$Gender) #age as continuous
variable
summary(DANVAlonelinessfreq_happy)

#Different age groups #age groups are 0=16-24, 1=25-34,2=35-64, 3=65+
#16-24 years
DANVAlonelinessfreq_YP <-
lm(data_full$total_DANVA_full~data_full$Loneliness_freq_zscore*data_full$Condi
tion+data_full$Gender,
                    subset=data_full$Age_groups==0)
summary(DANVAlonelinessfreq_YP) #Specific replication of age group used for
previous studies
lowDANVAlonelinessfreq_YP <-
lm(data_full$DANVA_lowintensity~data_full$Loneliness_freq_zscore*data_full$Co
ndition+data_full$Gender,
                         subset=data_full$Age_groups==0)
summary(lowDANVAlonelinessfreq_YP)
DANVAlonelinessfreq_fear_YP <-
lm(data_full$DANVA_fear_lowinstensity~data_full$Loneliness_freq_zscore*data_fu
ll$Condition+data_full$Gender,
                       subset=data_full$Age_groups==0) #age as continuous variable
summary(DANVAlonelinessfreq_fear_YP)
DANVAlonelinessfreq_anger_YP <-
lm(data_full$DANVA_anger_lowinstensity~data_full$Loneliness_freq_zscore*data_
full$Condition+data_full$Gender,
                       subset=data_full$Age_groups==0) #age as continuous variable
summary(DANVAlonelinessfreq_anger_YP)
DANVAlonelinessfreq_sad_YP <-
lm(data_full$DANVA_sad_lowinstensity~data_full$Loneliness_freq_zscore*data_fu
ll$Condition+data_full$Gender,
                      subset=data_full$Age_groups==0) #age as continuous variable
summary(DANVAlonelinessfreq_sad_YP)
DANVAlonelinessfreq_happy_YP <-
lm(data_full$DANVA_happy_lowinstensity~data_full$Loneliness_freq_zscore*data
_full$Condition+data_full$Gender,
                       subset=data_full$Age_groups==0) #age as continuous variable
summary(DANVAlonelinessfreq_happy_YP)

#25-34 years
DANVAlonelinessfreq_2534 <-
lm(data_full$total_DANVA_full~data_full$Loneliness_freq_zscore*data_full$Condi
tion+data_full$Gender,
                    subset=data_full$Age_groups==1)
```

```
summary(DANVAlonelinessfreq_2534)
lowDANVAlonelinessfreq_2534 <-
lm(data_full$DANVA_lowintensity~data_full$Loneliness_freq_zscore*data_full$Co
ndition+data_full$Gender,
                          subset=data_full$Age_groups==1)
summary(lowDANVAlonelinessfreq_2534)
#additional exploration - low intensity emotions separately
lowDANVAlonelinessfreq_fear_2534 <-
lm(data_full$DANVA_fear_lowinstensity~data_full$Loneliness_freq_zscore*data_fu
ll$Condition+data_full$Gender,
                              subset=data_full$Age_groups==1)
summary(lowDANVAlonelinessfreq_fear_2534)
lowDANVAlonelinessfreq_anger_2534 <-
lm(data_full$DANVA_anger_lowinstensity~data_full$Loneliness_freq_zscore*data_
full$Condition+data_full$Gender,
                              subset=data_full$Age_groups==1)
summary(lowDANVAlonelinessfreq_anger_2534)
lowDANVAlonelinessfreq_sad_2534 <-
lm(data_full$DANVA_sad_lowinstensity~data_full$Loneliness_freq_zscore*data_fu
ll$Condition+data_full$Gender,
                             subset=data_full$Age_groups==1)
summary(lowDANVAlonelinessfreq_sad_2534)
lowDANVAlonelinessfreq_happy_2534 <-
lm(data_full$DANVA_happy_lowinstensity~data_full$Loneliness_freq_zscore*data
_full$Condition+data_full$Gender,
                              subset=data_full$Age_groups==1)
summary(lowDANVAlonelinessfreq_happy_2534)

#35-64 years
DANVAlonelinessfreq_3564 <-
lm(data_full$total_DANVA_full~data_full$Loneliness_freq_zscore*data_full$Condi
tion+data_full$Gender,
                        subset=data_full$Age_groups==2)
summary(DANVAlonelinessfreq_3564)
lowDANVAlonelinessfreq_3564 <-
lm(data_full$DANVA_lowintensity~data_full$Loneliness_freq_zscore*data_full$Co
ndition+data_full$Gender,
                           subset=data_full$Age_groups==2)
summary(lowDANVAlonelinessfreq_3564)
DANVAlonelinessfreq_fear_3564 <-
lm(data_full$DANVA_fear_lowinstensity~data_full$Loneliness_freq_zscore*data_fu
ll$Condition+data_full$Gender,
                             subset=data_full$Age_groups==2) #age as continuous variable
summary(DANVAlonelinessfreq_fear_3564)
DANVAlonelinessfreq_anger_3564 <-
lm(data_full$DANVA_anger_lowinstensity~data_full$Loneliness_freq_zscore*data_
full$Condition+data_full$Gender,
                              subset=data_full$Age_groups==2) #age as continuous variable
summary(DANVAlonelinessfreq_anger_3564)
```

```
DANVAlonelinessfreq_sad_3564 <-
lm(data_full$DANVA_sad_lowinstensity~data_full$Loneliness_freq_zscore*data_full$Condition+data_full$Gender,
                         subset=data_full$Age_groups==2) #age as continuous variable
summary(DANVAlonelinessfreq_sad_3564)
DANVAlonelinessfreq_happy_3564 <-
lm(data_full$DANVA_happy_lowinstensity~data_full$Loneliness_freq_zscore*data_full$Condition+data_full$Gender,
                          subset=data_full$Age_groups==2) #age as continuous variable
summary(DANVAlonelinessfreq_happy_3564)

#65+ years
DANVAlonelinessfreq_65plus <-
lm(data_full$total_DANVA_full~data_full$Loneliness_freq_zscore*data_full$Condition+data_full$Gender,
                     subset=data_full$Age_groups==3)
summary(DANVAlonelinessfreq_65plus)
lowDANVAlonelinessfreq_65plus <-
lm(data_full$DANVA_lowintensity~data_full$Loneliness_freq_zscore*data_full$Condition+data_full$Gender,
                        subset=data_full$Age_groups==3)
summary(lowDANVAlonelinessfreq_65plus)
DANVAlonelinessfreq_fear_65plus <-
lm(data_full$DANVA_fear_lowinstensity~data_full$Loneliness_freq_zscore*data_full$Condition+data_full$Gender,
                          subset=data_full$Age_groups==3) #age as continuous variable
summary(DANVAlonelinessfreq_fear_65plus)
DANVAlonelinessfreq_anger_65plus <-
lm(data_full$DANVA_anger_lowinstensity~data_full$Loneliness_freq_zscore*data_full$Condition+data_full$Gender,
                          subset=data_full$Age_groups==3) #age as continuous variable
summary(DANVAlonelinessfreq_anger_65plus)
DANVAlonelinessfreq_sad_65plus <-
lm(data_full$DANVA_sad_lowinstensity~data_full$Loneliness_freq_zscore*data_full$Condition+data_full$Gender,
                         subset=data_full$Age_groups==3) #age as continuous variable
summary(DANVAlonelinessfreq_sad_65plus)
DANVAlonelinessfreq_happy_65plus <-
lm(data_full$DANVA_happy_lowinstensity~data_full$Loneliness_freq_zscore*data_full$Condition+data_full$Gender,
                          subset=data_full$Age_groups==3) #age as continuous variable
summary(DANVAlonelinessfreq_happy_65plus)
```
